# Computational investigation of cobalt and copper bis (oxothiolene) complexes as an alternative for olefin purification

**DOI:** 10.1007/s00894-020-04445-x

**Published:** 2020-07-10

**Authors:** Dušan N. Sredojević, Rajesh K. Raju, Salvador Moncho, Milivoj R. Belić, Edward N. Brothers

**Affiliations:** 1 Science Program, Texas A&M University at Qatar, Education City, Doha, Qatar; 2 Institute of Nuclear Sciences, Vinča, University of Belgrade, P.O. Box 522, Belgrade, 11001 Serbia

**Keywords:** Olefin purification, Density functional theory, Reaction mechanisms, Electro-catalyst

## Abstract

Considering that olefins present a large volume feedstock, it is reasonable to expect that their purification is industrially critical. After the discovery of the nickel bis (dithiolene) complex Ni(S_2_C_2_(CF_3_)_2_)_2_ that exhibits electro-catalytic activity with olefins but tends to decompose by a competitive reaction route, related complexes have been explored experimentally and theoretically. In this paper, a computational examination is performed on differently charged cobalt and copper bis (oxothiolene) complexes [M (OSC_2_(CN)_2_)_2_] to test their potential applicability as the catalysts for olefin purification, using the simplest olefin, ethylene. Possible reaction pathways for ethylene addition on these complexes were explored, to determine whether some of these candidates can avoid the reaction route that leads to decomposition, which is distinctive from the nickel complex, and to form stable adducts that can subsequently release ethylene by reduction. Our calculations suggest that the neutral cobalt complex might be an alternative catalyst, because all its forms can bind ethylene to produce stable interligand adducts with moderate to low activation barriers, rather than to form intraligand adducts that lead to decomposition. The calculations also predict that these interligand adducts are capable of releasing ethylene upon reduction. In addition, it can produce the desired interligand adducts following two different reaction pathways, assigned as the direct and the indirect, with no need for anion species as co-catalysts, which is crucial for the nickel complex. Thus, the olefin purification process could be much simpler by using this catalyst.

**Electronic supplementary material:**

The online version of this article (10.1007/s00894-020-04445-x) contains supplementary material, which is available to authorized users.

## Introduction

**Scheme 1:**
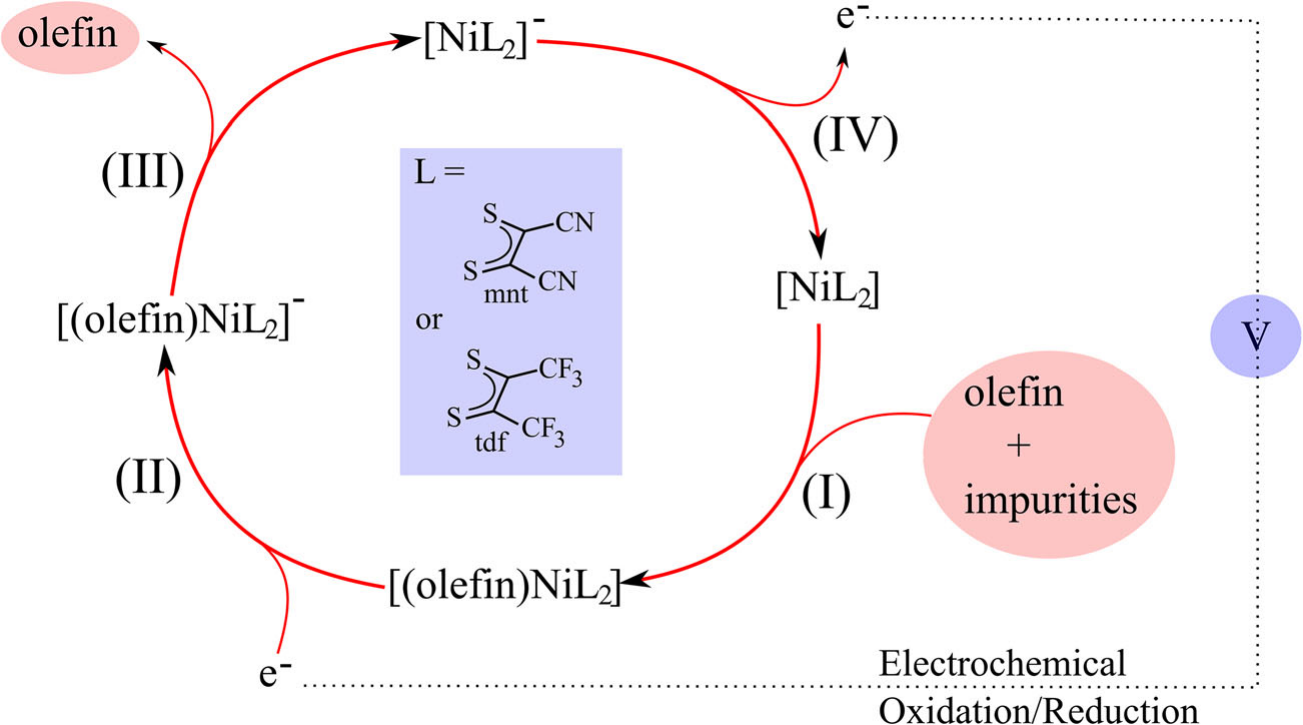
Reaction cycle for olefin purification with nickel bis (dithiolene) complexes acting as electro-catalysts [5]

Separation of olefins from petrochemical feedstock is an important process in the chemical industry and has attracted considerable attention for decades [1, 2]. Pure olefins are necessary as precursors to produce important chemicals and polymers, but their petrochemical origin makes them appear in mixtures with several other molecules. Thus, a suitable method for their purification is required as an alternative to the currently used cryogenic distillation that is energy-intensive, and, thus, expensive [3]. Many attempts have been made to develop more cost-effective methods, such as those based on transition metal complexes [4]. However, these complexes undergo deactivation in the presence of common impurities (H_2_O, CO, H_2_S, acetylene, etc.). A landmark in this search was the work of Wang and Stiefel in 2001, which reported an effective electrochemical system for olefin purification based on the nickel bis (dithiolene) complexes (Ni(S_2_C_2_R_2_)_2_, *R* = CF_3_ and CN (**1**) which reversibly and selectively react with the olefins and avoid poisoning by common impurities [5]. The proposed electro-catalytic reaction includes four successive steps: (I) the formation of the olefin adduct [(olefin)NiL_2_]; (II) the electrochemical reduction to produce [(olefin)NiL_2_]^−^, (III) rapid releases of the olefin to regenerate [NiL_2_]^−^; (IV) the oxidation of the anionic complex [NiL_2_]^−^ (**1**^−^) to the neutral species [NiL_2_] (**1**) (Scheme 1). X-ray crystallographic structural analysis has confirmed that the olefin adducts, formed in step I, possess a C–C sequence that bridges the two sulfur atoms from the opposite dithiolene ligands, named the interligand adduct [6, 7].

Several papers demonstrated that the metal bis (dithiolene) complexes display high reactivity toward strained and cyclic alkenes, forming cyclo-addition products [6, 8–19]. However, a direct pathway that leads to the formation of the desired *cis*-interligand adduct is symmetry-forbidden, which is in line with the Woodward-Hoffman addition rules [20]. Otherwise, this adduct can be formed through a twisted (pseudo-tetrahedral) intermediate, which is formed in the first step of the reaction, avoiding constraints imposed by orbital symmetry. This was proposed in 2002 by Fan and Hall and summarized as a two-step mechanism that begins with the olefin addition followed by isomerization [20].

**Scheme 2:**
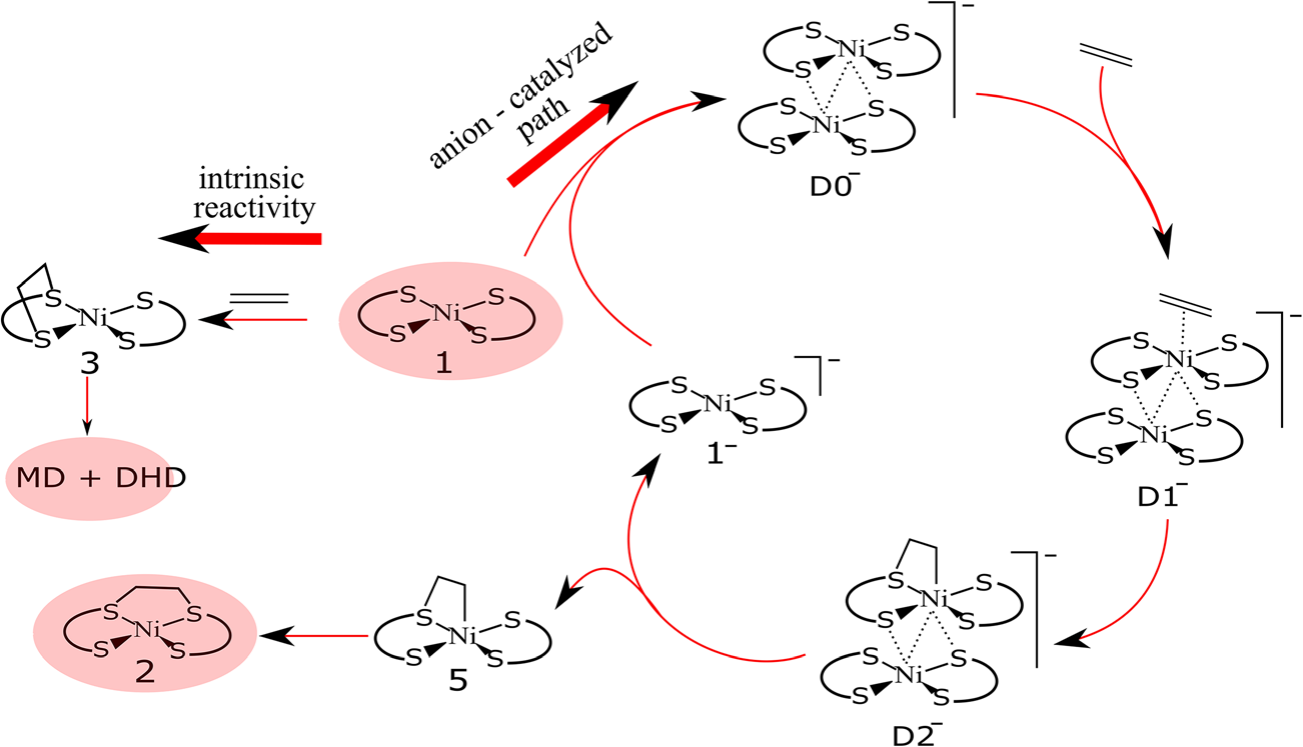
The anion-catalyzed reaction competes with the intrinsic reactivity of **1** [21]

Experimental results reported in 2006 suggested that the interligand adduct can only be formed in the presence of an anionic complex [NiL_2_]^−^ (**1**^−^) [7]. Otherwise, in the absence of the reduced complex, the main products of the reaction between [NiL_2_] (**1**) and olefins are a series of decomposition species such as substituted dihydrodithiin (DHD) and metal-containing products (MD). These findings led to the revision of the mechanism of 2002. The first remark was that the reaction of **1** with ethylene, in the absence of anion **1**^−^, leads to the formation of the intraligand adduct (symmetry-allowed), which further decomposes to produce experimentally detected DHD and MD species. In this isomer, the ethylene molecule forms a C–C bridge between the two sulfur atoms on the same dithiolene ligand, forming a carbon-sulfur ring. The *cis*-interligand adduct can be formed via a complicated four-step process that includes different bimetallic species (**1/1**^−^) and the formation of an intermediate with a Ni-S coordination of the ethylene (Scheme 2). The revised mechanism was proposed by Hall and co-workers as a result of combined experimental and theoretical studies, which highlighted the crucial role of the reduced anionic complex acting as a co-catalyst [21, 22].

Several theoretical studies have been published recently, with the aim of finding catalysts for olefin purification that perform better than the original nickel bis (dithiolene) complex [23–28]. In one of them, a set of novel complexes was generated by changing the nickel atom with cobalt or copper atoms and by varying the overall charge of the complex [23]. It turned out that the neutral cobalt complex [Co(S_2_C_2_(CF_3_)_2_)_2_] had the best performance for olefin purification among the tested complexes because it combined low activation barriers to form the appropriate olefin adducts without the need of a co-catalyst and to release the olefin upon reduction. Another study revealed that nickel complexes with oxothiolene ligands showed enhanced properties compared to the parent complex [24]. The two isomers of nickel bis (oxothiolene) (*cis* and *trans*) showed higher reaction selectivity toward the formation of desired interligand adducts, which are in turn much more thermodynamically stable than the intraligand adduct. Moreover, both isomers can catalyze the desired reaction with low activation barriers (8–9 kcal/mol) without the anion as a co-catalyst [22]. The most intriguing results have emerged from our previous DFT study exploring the possible reaction pathways between ethylene and nickel bis (diselenolene) complexes [25]. Surprisingly, these complexes do not decompose through intraligand adducts, because the calculated model for decomposition predicts the barriers of very high energy (38–45 kcal/mol).

In this work, we extend our previous investigations, especially those based on the cobalt and copper bis (dithiolene) and nickel bis (oxothiolene) series of complexes [23, 24]. Thus, we combined these structures to study the related cobalt and copper bis (oxothiolene) complexes (*cis* and *trans*-isomers), to examine their reactivity with ethylene. The aim is to obtain a complex that improves the performance of previous catalysts: providing low barriers, preventing the formation of the intraligand adduct (or its decomposition), and reversibly binding ethylene, while excluding the need for an anion as the co-catalyst.

## Materials and methods

All calculations have been done using the Gaussian 09 suite of programs [29]. We chose the ωB97XD [30] density functional for all calculations, because it was established that this functional produces results that are in good agreement with those obtained with the accurate coupled cluster calculations (CCSD) in a simplified model system, Ni(S_2_C_2_H_2_)_2_ [31]. This functional contains both long-range exchange and empirical dispersion corrections that are crucial for the accurate predictions of reaction barriers and for modeling the systems with weak interactions. The Pople all-electron 6–31++G** basis set was specified for all atoms [32]. The geometries of all species were optimized in the gas phase and we used a vibrational analysis to determine the nature of all intermediates (no imaginary frequencies) and transition states (with only one imaginary frequency). Intrinsic reaction coordinate calculations (IRC) were applied in some cases to confirm that a transition state connects the appropriate intermediates [33]. Additional calculations were performed for all the species, to test the stability of DFT wave functions, i.e., to check that the obtained electron density corresponds to the lowest energy electron distribution [34, 35]. For those species that we could not optimize a geometry corresponding the stable solution (very few cases), we used the equation proposed by Yamaguchi’s broken-spin-symmetry procedure to estimate the energy of the spin-purified low spin state (^LS^E) [36, 37]. This includes the calculation of energies of the broken symmetry solution (^BS^E) and the high spin coupled state (^HS^E) in a geometry optimized using an unstable density, according to the following formula:

$$ {\mathrm{LS}}_{\mathrm{E}}=\frac{{\mathrm{BS}}_{\mathrm{E}}\left({\mathrm{HS}}_{\left\langle {\mathrm{S}}^2\right\rangle }-{\mathrm{LS}}_{\left\langle {\mathrm{S}}^2\right\rangle}\right)-{\mathrm{HS}}_{\mathrm{E}}\left({\mathrm{BS}}_{\left\langle {\mathrm{S}}^2\right\rangle }-{\mathrm{LS}}_{\left\langle {\mathrm{S}}^2\right\rangle}\right)}{{\mathrm{HS}}_{\left\langle {\mathrm{S}}^2\right\rangle }-{\mathrm{BS}}_{\left\langle {\mathrm{S}}^2\right\rangle }} $$

The solvation effects, with chloroform as a solvent, were calculated using the SMD method, with geometries previously optimized in the gas phase [38]. The free energies in solution (Δ*G*_sol_) were calculated by adding solvation energies to the gas-phase relative free energies. The corrected solvent-free energy values will be used in discussion throughout the paper, unless otherwise stated. All 3D molecular structures shown in this paper were drawn using the CYL*view* software for molecular visualization [39].

## Results and discussion

An ideal catalyst for olefin purification should prevent decomposition by imposing high energy requirements for the formation of the intraligand adduct **3**, which is known for tendency to decompose. Furthermore, it should reversibly bind ethylene through a stable intermediate (such as the interligand adduct **2** or the twisted intermediate **2y**) with lower barriers than the previous alternatives (such as the original nickel bis (dithiolene) complex) in a pathway that does not require the anion as a co-catalyst. Keeping these demands, we followed the systematic search for alternative catalysts that were considered in our previous work. Compared to the original **1**_**_Ni_SS**_ complex, the two isomers of **1**_**_Ni_OS**_ (*cis*/*trans*) [24] and **1**_**_Co_SS**_ [23] complexes show higher thermodynamic selectivity toward the formation of the most desired *cis*-interligand adduct **2**. Thus, in this paper, we examined the related [Co (OSC_2_(CN)_2_)_2_]^*n*^ (*n* = 0, − 1) and [Cu (OSC_2_(CN)_2_)_2_]^*n*^ (*n* = + 1, 0, − 1, − 2) complexes and their reactions with ethylene; OSC_2_(CN)_2_– oxothiolene, analogous of S_2_C_2_(CN)_2_. We start with the thermodynamic prediction of the formation of relevant adducts (**2**, **2y**, and **3**), which is briefly presented in the next section. Selected complexes from the next section with favorable thermodynamics, in accordance with above requirements, have been tested in reaction with ethylene, by locating the transition states (reaction barriers) to explore reaction profiles.

### Thermodynamic results for the reactions of relevant complexes with ethylene

**Scheme 3:**
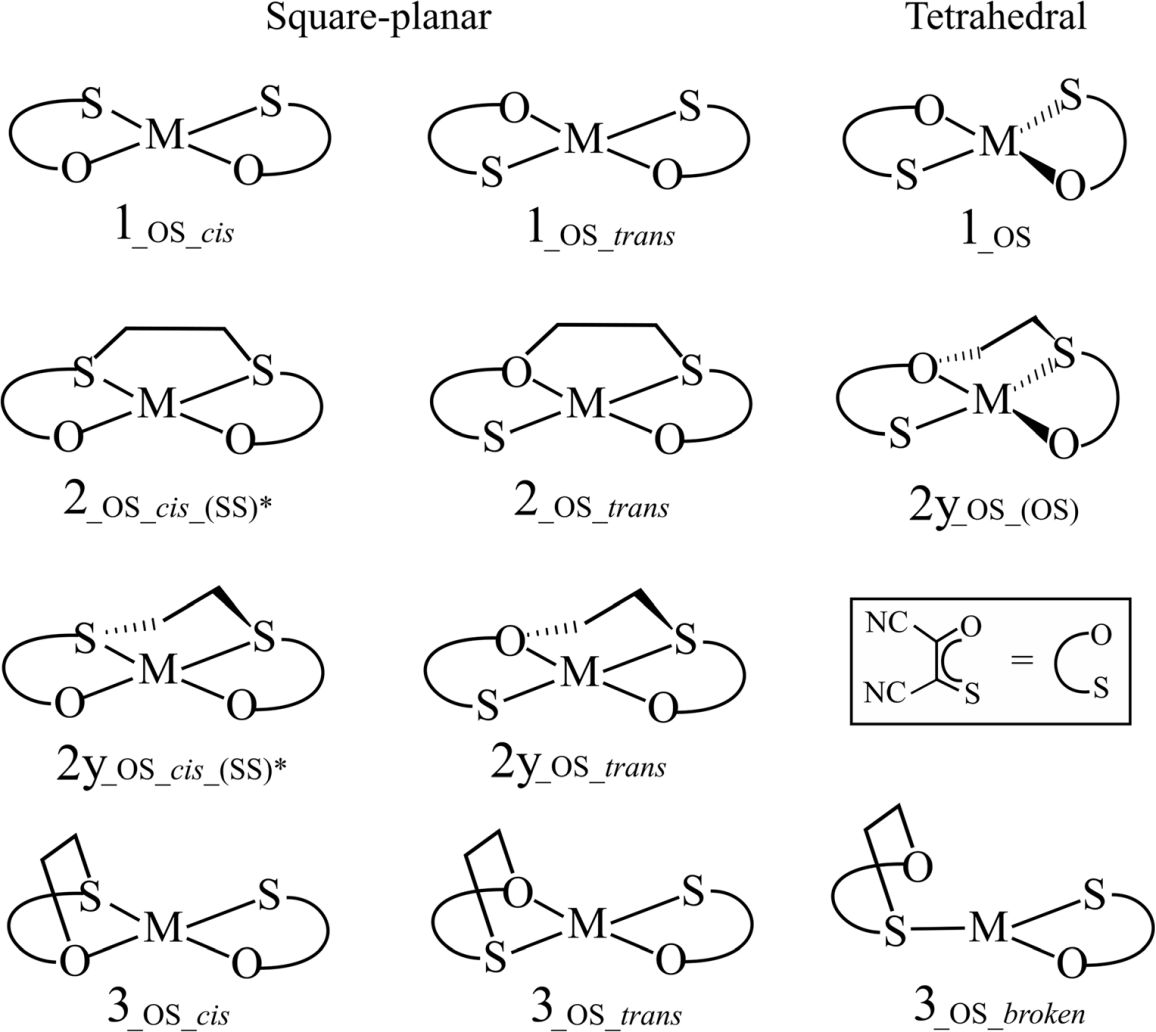
The graphical presentation of the analyzed cobalt and copper complexes as well as their ethylene adducts. **2**, **2y**, and **3** refer to the interligand, twisted intermediate, and intraligand adducts, respectively. “*” denotes that the second binding mode is also possible (SS vs. OO)

Thermodynamic stability of the usual products (intra and interligand; Scheme 3) for different cobalt and copper bis (oxothiolene) complexes are summarized in Table 1, compared with previously reported results for nickel, cobalt and copper bis (dithiolene) [23], and nickel bis (oxothioloene) complexes [24]. The nature of the ligand is shown in the subscript after the designation of the metal, for example, **1**_**_Ni_SS**_ denotes nickel bis (dithiolene), while **1**_**_Co_OS**_ denotes the cobalt bis (oxothiolene) complex. It should be noted that all bis (oxothiolene) complexes have attached CN groups, while bis (dithiolene) complexes are in CF_3_–substituted system; however, our previous studies in similar complexes suggest that both groups lead to very similar energies [21, 22, 25, 40].

**Table 1:** Calculated free energies in solvent (CHCl_3_) for the nickel, cobalt, and copper bis (dithiolene) as well as bis (oxothiolene) complexes

Complex	Planar	Tetrahedral	2y	2	3
^1^1__Ni_SS_ [21, 22]	0.0	–	− 16.1	− 17.8	− 14.8
^1^1__Ni_OS_*cis*_ [24]	0.0	–	− 8.6	− 19.1	3.9
^1^1__Ni_OS_*trans*_ [24]	0.0	–	–	− 7.5	5.2
^2^1__Co_SS_ [23]	0.0	–	− 22.3	− 25.6	− 13.3
^4^1__Co_OS_	–	0.0	− **27.2**_(SS)_ − 8.1_(OO)_ − 18.2 _(OS)_	− 24.2 _(SS)_	− 10.5^a^
				− 20.4 _(OS)_	
^2^1__Co_OS_*cis*_	6.3	–	0.3 _(OS)_ 2.9 _(OO)_	− 13.5 _(SS)_	12.2
^2^1__Co_OS_*trans*_	4.6	–	− 14.6_(SS)_	− 2.1 _(OS)_	13.8
^1^1__Co_OS_*cis*_^−^	6.6	–	22.1_(SS)_ 43.0 _(OO)_	25.7	44.1^a^
^1^1__Co_OS_*trans*_^−^	8.5	–	32.2	32.8	44.1^a^
^3^1__Co_OS_^−^	–	0.0	7.1_(SS)_ 26.7 _(OO)_	12.3	41.2
^1^1__Cu_SS_^-^ [23]	0.0	–	− 9.5	− 3.3	− 1.5
^1^1__Cu_OS_^*+*^	–	0.0	− 17.5_(SS)_ − 22.2 _(OO)_	− 17.9	− 32.0^a^
^3^1__Cu_OS_*cis*_^*+*^	3.1	–	− 21.4_(SS)_ − **25.6**_(OO)_	− 20.4	− 24.5
^3^1__Cu_OS_*trans*_^*+*^	3.4	–	− 21.6	− 23.3	− 23.0^a^
^2^1__Cu_OS_*cis*_	0.4	–	− 15.2_(SS)_ − 7.0 _(OO)_	− 20.1	− 20.4^a^
^4^1__Cu_OS_*cis*_	0.0	–	32.9_(SS)_ 33.3 _(OO)_	29.9	19.8^a^
^2^1__Cu_OS_*trans*_	0.5	–	− 13.2	− 13.5	− 20.9^a^
^4^1__Cu_OS_*trans*_	0.0	–	37.2	27.5	19.1
^1^1__Cu_OS_*cis*_^−^	0.8	0.8	− 17.4_(SS)_ − 5.9 _(OO)_	− 13.4	0.1^a^
^3^1__Cu_OS_^−^	–	0.0	25.4_(SS)_ 27.8 _(OO)_	35.5	33.5
^2^1__Cu_OS_^2−^	–	0.0	39.2	44.2	36.8^a^
^4^1__Cu_OS_^2−^	–	55.7	82.0	85.7	79.3^a^

Denotations inside the parenthesis (SS, OS, and OO) indicate that ethylene binds to the sulfur atoms, oxygen and sulfur, and oxygen atoms of different chelates, respectively

^a^Broken geometry with three atoms coordinated to the metal atom

Among the neutral **1**_**_Ni_OS**_ complexes, the *cis* isomer exhibits more favorable thermodynamics compared to the *trans* isomer when ethylene is bound to the sulfur atoms. However, the coordination of ethylene in the oxygen side of the *cis* isomer is significantly less stable [24]. It should be noted that for the *cis*-isomer, O-binding ethylene adducts only exist in a twisted geometry **2y**. For the selection of the total charge of the complex, we based our selection in our previous results for bis (dithiolene) complexes. Only the neutral **1**_**_Co_SS**_ complex was regarded as a potential catalyst for olefin purification, while negatively charged complexes display unfavorable thermodynamics [23]. Having that in mind, we investigated only neutral and mono-anionic cobalt bis (oxothiolene) (**1**_**_Co_OS**_^**0/1−**^) complexes. Since previous results for **1**_**_Cu_SS**_ complexes showed that for positively charged and neutral complexes, the intraligand adducts **3** are more stable than inerligand adducts **2**; the only alternative catalyst could be the mono-anionic **1**_**_Cu_SS**_^**−**^ complex, whose reaction with ethylene is predicted to be almost thermodynamically neutral. In this section, the results for the **1**_**_Cu_OS**_^***n***^ (*n* = + 1, 0, − 1, − 2) complexes are also presented. For all the copper and cobalt bis (oxothiolenes) in this study, two different spin states were considered: the lowest (singlet or doublet depending of the number of electrons) and the first higher spin state (triplet or quartet).

### Cobalt bis (oxothiolene) complexes

The most stable adduct for the neutral **1**_**_Co_OS**_ complex is ^**4**^**2y** (tetrahedral), in SS-binding mode, which is more stable than ^**4**^**2** (planar) by 3 kcal/mol (Table 1). In contrast, for the OS-binding mode, the *cis*-interligand adduct ^**4**^**2** (planar) is more stable than ^**4**^**2y** (tetrahedral) by 2.2 kcal/mol (Table 1). As in similar complexes, the least stable adduct is the one associated with the OO-binding mode, which can only exist in twisted geometry ^**4**^**2y** (− 8.1 kcal/mol). Nevertheless, the ^**4**^**2**_**_Co_OS**_ adducts in SS and OS binding modes are more stable than adduct ^**4**^**3**_**_Co_OS**_, by 13.7 and 9.9 kcal/mol, respectively. That difference is much larger than for the **1**_**_Ni_SS**_ system, and comparable for those in the **1**_**_Co_SS**_ system, suggesting a higher selectivity toward the formation of desired interligand products. In the doublet state, the ^**2**^**1**_**_Co_OS_*cis***_ complex, in SS-binding mode, shows the biggest difference between relative stability of adducts **2** and **3**, 25.7 kcal/mol, larger than for its nickel analog ^**1**^**1**_**_Ni_OS_*cis***_ (23 kcal/mol) [24]. For the *trans*-isomer (^**2**^**1**_**_Co_OS_*trans***_), the difference is moderate (15.9 kcal/mol) and the ^**2**^**2**_**_Co_OS_*trans***_ adduct is not quite stable (− 2.1 kcal/mol). Nevertheless, it can be concluded that, from thermodynamic values, the neutral **1**_**_Co_OS**_ complex, in both spin states, might be regarded as an alternative catalyst (Table 1; underlined). The anionic cobalt complex (**1**_**_Co_OS_*cis***_^**−**^), which is isoelectronic to the neutral **1**_**_Ni_SS**_ and **1**_**_Ni_OS**_, favors the triplet state, as well as its ethylene adducts. However, ethylene adducts on anionic cobalt are thermodynamically unfavorable, which might be excluded as candidates for the olefin separation process. The reason for this can be attributed to the fact that ethylene plays a role of nucleophile in these reactions. On the other hand, the instability of anionic adducts, in both spin states, supports the claim of the neutral cobalt complex, because the ethylene could be released from these adducts after reduction.

### Copper bis (oxothiolene) complexes

Among the copper complexes, only the mono-anionic complex (^**1**^**1**_**_Cu_OS**_^***−***^) might be regarded as an alternative catalyst according to our two main requirements (Table 1; underlined). First, due to the favorable thermodynamics of both interligand adducts (^**1**^**2y**_**_Cu_OS_*cis***_^***−***^, ^**1**^**2**_**_Cu_OS_*cis***_^***−***^ by 17.4 and 13.4 kcal/mol, respectively) compared to the intraligand ^**1**^**3**_**_Cu_OS_*cis***_^***−***^ (isoenergetic with the reactants). Second, because their adducts become unstable upon reduction, **2y**_**_Cu_OS_*cis***_^***2−***^, **2**_**_Cu_OS_*cis***_^***2−***^, **3**_**_Cu_OS_*broken***_^***2−***^ are at least 35 kcal/mol less stable than the ethylene release products). Despite the mono-anionic copper complex **1**_**_Cu_OS**_^***−***^ prefers triplet state, this spin state shows very unfavorable thermodynamics for the ethylene bindings. However, singlet-state complex ^**1**^**1**_**_Cu_OS**_ is accessible (only slightly less stable than the triplet ~ 0.8 kcal/mol) and can adopt both planar (only as the *cis*-isomer) and tetrahedral geometries. On the other hand, cationic and neutral complexes can be discarded because the stability of intraligand, decomposing adducts (like the interligand or significantly more stable). On the other hand, the double-anionic complex **1**_**_Cu_OS**_^***2−***^ has very unfavorable thermodynamics for ethylene binding in both spin states, probably because it is too electron-rich to act as an electrophile. This makes it not feasible as a catalyst, but contributes to the feasibility of the monoanionic complex, because their adducts can be released upon reduction.

### The relative stability of adducts

The stability of ethylene adducts with the series of isoelectronic species decreases from that with cationic copper (**1**_**_Cu_OS_*cis/trans***_^**+**^), through neutral nickel (**1**_**_Ni_OS_*cis/trans***_) [24], to anionic cobalt (**1**_**_Co_OS_*cis/trans***_^**−**^) complexes (see Table 1). There are two explanations for this trend. First, considering that the ethylene acts as a nucleophile in these reactions, it is reasonable that its addition to the positively charged copper complex is more favorable than to the neutral nickel complex, which is in turn, more favorable than to the negatively charged cobalt complex. The second explanation can be given through the analysis of the frontier molecular orbitals (FMO) of relevant species. Thus, we compared the energy differences (ΔE) between LUMO orbitals of different *cis*-isomers, which appear to be similar for all three complexes, and HOMO orbital of ethylene (Table 2). It is evident from Table 2 that Δ*E* increases in the following order: **1**_**_Cu_OS_*cis***_^***+***^ < **1**_**_Ni_OS_*cis***_ < **1**_**_Co_OS_*cis***_^−^, thereby reducing the binding abilities of certain complex to ethylene (Table 2). In respect to the series of isocharged complexes (^**1**^**1**_**_Ni_OS_*cis***_, ^**2**^**1**_**_Co_OS_*cis***_, ^**2**^**1**_**_Cu_OS_*cis***_), Δ*E* values are similar in all three cases. The higher deviation in stability is noticed for adduct ^**2**^**3**_**_Cu_OS**_, which has three-coordinated broken geometry.

**Table 2:** Energies (eV) of frontier orbitals (FMO) for different complexes and ethylene

Complex	HOMO	LUMO	Δ*E*^a^
**C**_**2**_**H**_**4**_	− 9.50	2.58	
^**1**^**1**_**_Ni_OS_*cis***_	− 8.35	− 4.17	5.33
^**2**^**1**_**_Co_OS_*cis***_	− 8.88 (− 8.89)	− 3.28 (− 3.18)	6.22 (6.32)
^**1**^**1**_**_Co_OS_*cis***_^**−**^	− 7.39	− 1.35	8.15
^**3**^**1**_**_Cu_OS_*cis***_^***+***^	− 9.99 (− 11.55)	− 5.45 (− 5.46)	4.05 (4.04)
^**2**^**1**_**_Cu_OS_*cis***_	− 8.74 (− 9.56)	− 1.88 (− 3.53)	7.62 (5.97)

All complexes in this table have planar geometries. For ^**2**^**1**_**_Co_OS_*cis***_, ^**3**^**1**_**_Cu_OS_*cis***_^***+***^, and ^**2**^**1**_**_Cu_OS_*cis***_ complexes, values inside the parentheses correspond to the βMO, whereas those outside the parentheses correspond to the αMO

^a^Δ*E* represents energy difference between complex’s LUMO and ethylene’s HOMO. Energies are given in eV and represent solvent-corrected MO energies that were calculated at ωB97X-D/6-31G(d,p) level. Smaller basis set was chosen to produce valence-like LUMOs

### Reaction mechanisms for cobalt bis (oxothiolene) complexes with ethylene

According to the thermodynamic results in Table 1, the neutral species (^**2**^**1**_**_Co_OS_*cis***_*,* ^**2**^**1**_**_Co_OS_*trans***_*,* and ^**4**^**1**_**_Co_OS**_) display promising properties for ethylene binding and might be regarded as a potential catalyst. Thus, the reaction mechanisms were investigated, by locating all possible intermediates and transition states, to estimate its potential from a kinetic point of view. Based on our previous research, two main types of mechanisms have been explored for neutral cobalt bis (oxothiolene) complexes. In the first mechanism, referred to as the “direct” pathway, ethylene adds directly to the sulfur (oxygen) of the same or opposite ligands, forming intraligand and interligand adducts, respectively. The second mechanism, referred as “indirect”, is characterized by the formation of an intermediate in the first step of the reaction, in which ethylene is bound to metal and ligator atoms. This intermediate can further transform into both interligand and intraligand adducts. In this section, both reaction pathways for all **1**_**_Co_OS**_ species (in both spin states) are reported. Furthermore, for each pathway, two possible approaches of ethylene were considered, depending if the initial coordination of the ethylene molecule is on sulfur or oxygen atoms (referred as S- and O-binding side). In general, the O-binding-side pathways are higher in energy and not competitive with the S-binding-side alternatives; for this reason, they are not detailed here and reported as Supporting Information. Although ^**4**^**1**_**_Co_OS**_ is tetrahedral (with no *cis/trans* isomerism), its reactivity is also described in two parts, divided into *cis* and *trans* based on the square-planar intermediates and products*.*

### Coupled-cluster calculations

The results for the neutral cobalt bis (oxothiolene) complexes show that the quartet-state ^**4**^**1**_**_Co_OS**_ (tetrahedral) complex is more stable than the double-state complexes ^**2**^**1**_**_Co_OS _*cis***_ and ^**2**^**1**_**_Co_OS_*trans***_ (planar) by 6.3 and 4.6 kcal/mol, respectively (Table 1). However, it has been proven that ωB97X-D functional can overestimate the energy gap between high- (HS) and low-spin (LS) states, in favor of the high spin state [23]. In order to check this and improve our estimation of the energy difference between complex in high (^**4**^**1**_**_Co_OS**_) and low (^**2**^**1**_**_Co_OS_*cis/trans***_) spin state, high-level calculations were done using CCSD(T) with the same basis set (6–31++G(d,p)) in a simplified model of general formula Co (OSC_2_H_2_)_2_ (**1**_**_Co_model**_). These calculations show that ^**2**^**1**_**_Co_model_*cis***_ (planar) is more stable than ^**4**^**1**_**_Co_model**_ (tetrahedral) by 2.1 kcal/mol (electronic energy), which is, in turn, more stable than ^**2**^**1**_**_Co_model_*trans***_ (planar) by 2.8 kcal/mol (electronic energy). In contrast, according to the ωB97X-D calculations, the most stable species is ^**4**^**1**_**_Co_model**_ (tetrahedral), which is more stable than ^**2**^**1**_**_Co_model_*cis***_ and ^**2**^**1**_**_Co_model_*trans***_ (planar) by 5.0 and 8.2 kcal/mol (electronic energy), respectively. Hence, ωB97X-D functional overestimates the high spin by at least 7 kcal/mol for **1**_**_Co_model**_ comparing to the CCSD(T). However, CCSD(T) calculations in the ethylene adducts show that ^**4**^**2**_**_Co_model_*cis***_ is more stable than ^**2**^**2**_**_Co_model_*cis***_ by 14.1 kcal/mol (electronic energy). The differences in stability between two spin states for various interligand adducts, according to the ωB97X-D functional, are in range from 11 to 18 kcal/mol, in favor of high-spin state. This indicates that ωB97X-D functional gives accurate prediction for the relative stability of the spin states in the interligand adducts (in comparison with the reactant complex). Hence, we consider that both spin states are similar in energy and both are accessible, and we will study the mechanism of the reaction in both spin states. Additional details on the CCSD(T) calculations can be found in the Electronic Supplementary Material (Fig. S1/S2 and Tables S1/S2).

### Energy profile of 1__Co_OS_ for quartet spin state

**Fig. 1:**
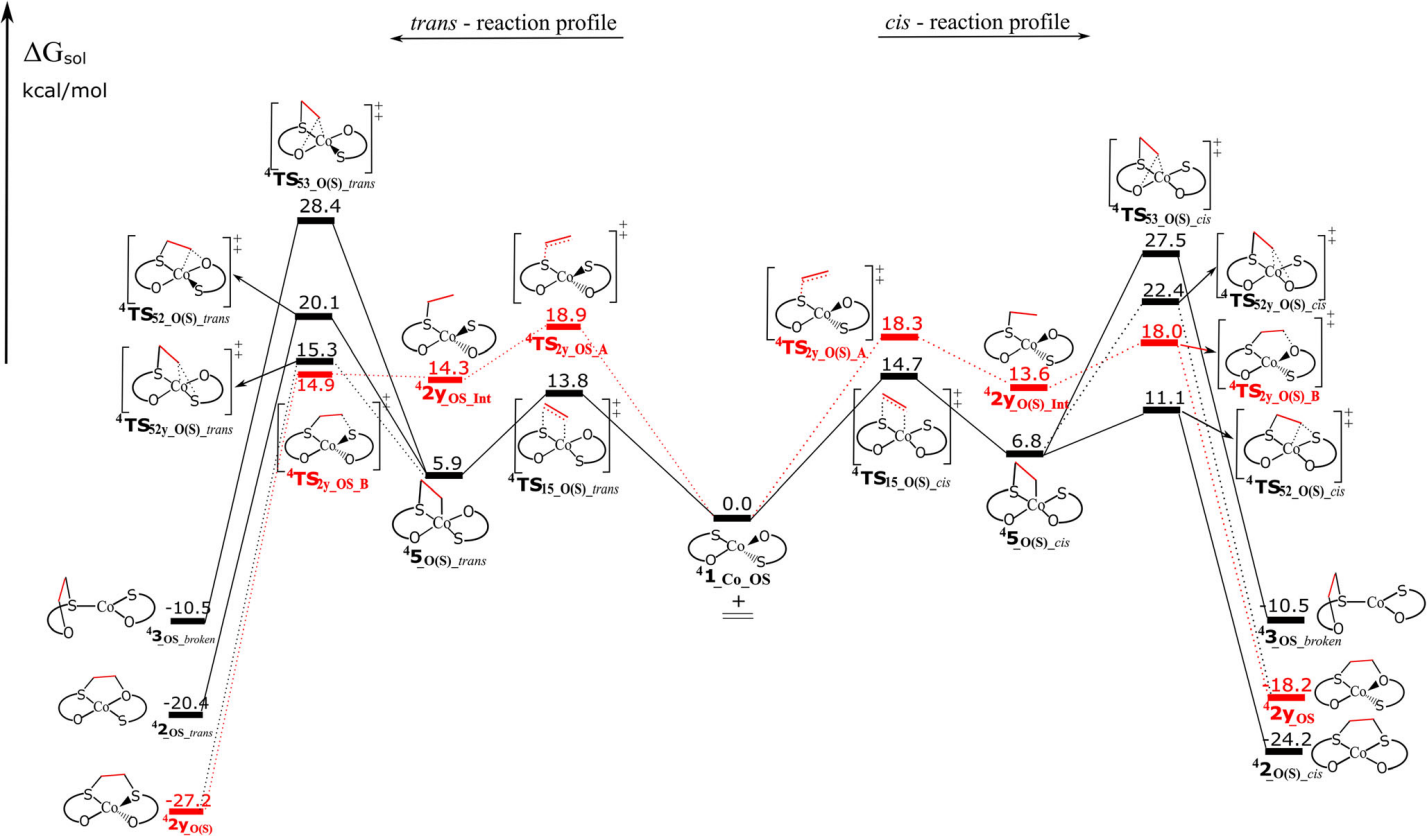
Calculated energy surfaces for the reaction of ^**4**^**1**_**_Co_OS**_ with ethylene, for the *cis*- and *trans*-reaction profiles, considering S-binding side. Dotted (red) lines represent direct and solid (black) lines represent indirect pathway. Energies in kcal/mol are the free energies in solvent (CHCl_3_)

**Fig. 2:**
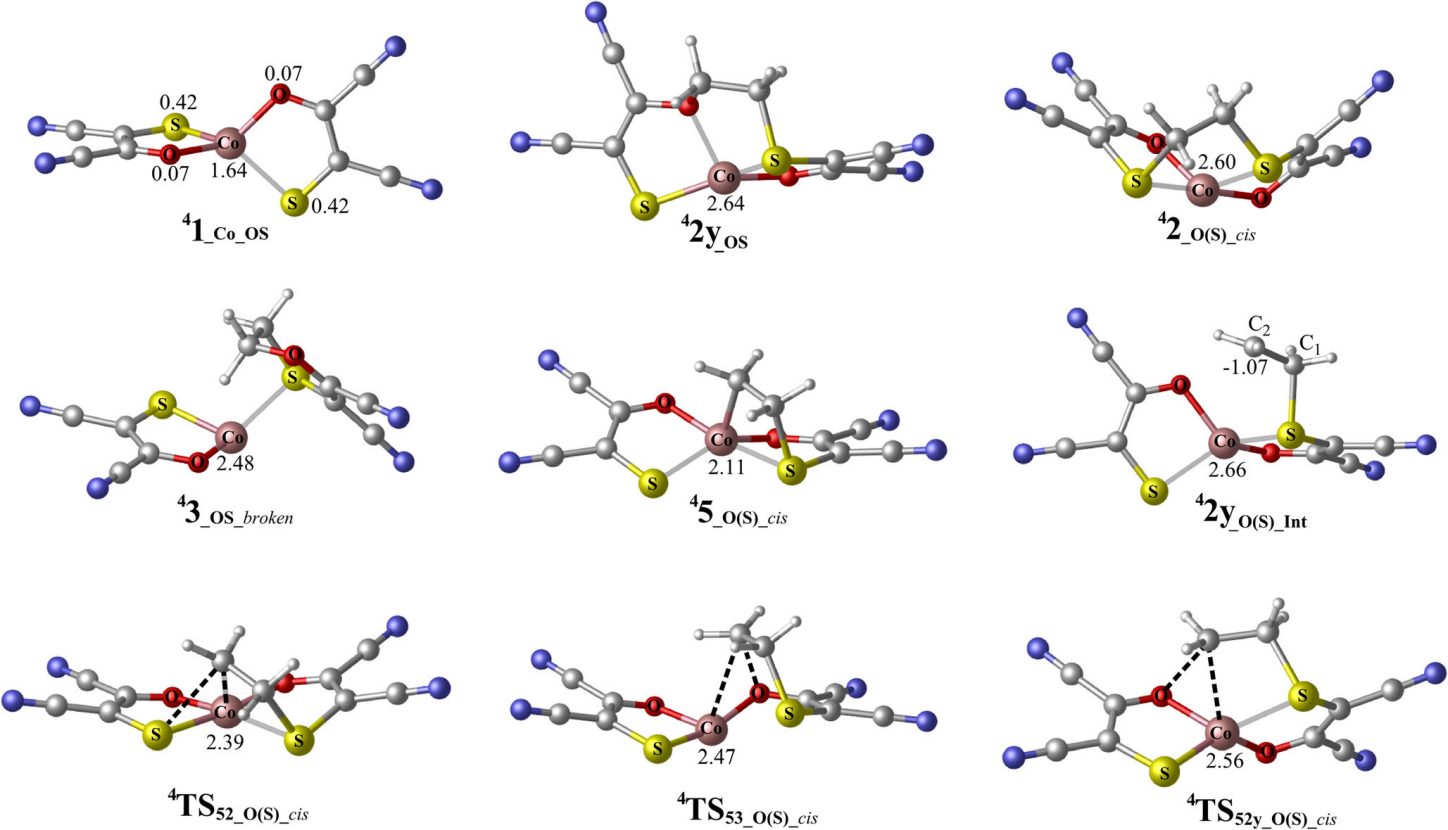
The optimized geometries for the selected species of *cis*-reaction profile that are included in Fig. 1. Values correspond to atomic spin densities. The geometries of selected species for *trans*-reaction profiles are presented in Electronic Supplementary Material (Fig. S3)

The quartet-state energy surface of ^**4**^**1**_**_Co_OS**_ and ethylene is presented in Fig. 1 (dotted red and solid black lines assign direct and indirect pathways, respectively). The optimized geometries of significant intermediates and transition states are presented in Fig. 2. In the *cis*-reaction profile, the direct pathway lead to a twisted, interligand product ^**4**^**2y**_**_OS**_ (tetrahedral) with an activation barrier of 18.3 kcal/mol for ^**4**^**TS**_**2y_O(S)_A**_. This reaction follows a stepwise pathway including the intermediate ^**4**^**2y**_**_O(S)_Int**_ (biradical species with one electron of β spin excess located on C_2_ carbon atom: Fig. 2). Formation of the intraligand adduct ^**4**^**3**_**_OS**_ via direct pathway was not located neither in a direct step nor successively. Along the indirect pathway for the *cis*-reaction profile, the intermediate ^**4**^**5**_**_O(S)_*cis***_, in which ethylene is connected to the Co and S atoms, can be formed via transition state ^**4**^**TS**_**15_O(S)_*cis***_ by crossing the barrier of 14.7 kcal/mol. This intermediate can further isomerize into different both interligand (planar ^**4**^**2**_**_O(S)_*cis***_ and tetrahedral ^**4**^**2y**_**_OS**_) and intraligand (^**4**^**3**_**_OS_*broken***_) adducts, by overcoming barriers of 4.3 kcal/mol (^**4**^**TS**_**52_O(S)_*cis***_), 15.6 kcal/mol (^**4**^**TS**_**52y_O(S)_*cis***_), and 20.7 kcal/mol (^**4**^**TS**_**53_O(S)_*cis***_), respectively. There is a high kinetic selectivity (16.4 kcal/mol compared with the intraligand isomer) towards the formation of desired interligand adduct ^**4**^**2**_**_O(S)_*cis***_. The indirect pathway that leads to ^**4**^**2**_**_O(S)_*cis***_ is kinetically and thermodynamically most favorable, with a rate-determining barrier of 14.7 kcal/mol.

About the *trans*-reaction profile (Fig. 1, left side), the direct pathway forms the twisted interligand adduct ^**4**^**2y**_**_O(S)**_ (tetrahedral) with an activation barrier of 18.9 kcal/mol (^**4**^**TS**_**2y_OS_A**_). Similarly, to the *cis* isomer, it is a stepwise process with the formation of the first C–S bond leading to the intermediate ^**4**^**2y**_**_OS_Int**_. This intermediate can further form the second C–S bond, via ^**4**^**TS**_**2y_OS_B**_, with an activation barrier of only 0.6 kcal/mol relative to ^**4**^**2y**_**_OS_Int.**_ Like the *cis*-reaction profile, the hypothetic formation of the intraligand adduct ^**4**^**3**_**_OS_*broken***_ through direct pathway was not located neither directly nor successively. The alternative indirect pathway includes the formation of an intermediate ^**4**^**5**_**_O(S)_*trans***_ (the ethylene is connected to the Co and S atoms) with an activation barrier of 13.8 kcal/mol (^**4**^**TS**_**15_O(S)_*trans***_). ^**4**^**5**_**_O(S)_*trans***_ can isomerize into three different adducts both interligand (^**4**^**2**_**_OS_*trans***_ and ^**4**^**2y**_**_O(S)**_) and intraligand (^**4**^**3**_**_OS_*broken***_, which can lead to the decomposition). However, the transition states that lead to the formation of interligand adducts (^**4**^**2**_**_OS_*trans***_ and ^**4**^**2y**_**_O(S)**_) are 8.3 and 13.1 kcal/mol lower in energy than one associated with the formation of the intraligand adduct, respectively. This indicates that indirect pathway drives the reaction toward the formation of the desired interligand adducts (^**4**^**2**_**_OS_*trans***_ and ^**4**^**2y**_**_O(S)**_) over the formation of broken adduct ^**4**^**3**_**_OS_*broken***_, both kinetically and thermodynamically.

The reactivity starting by an oxygen atom (O-binding side) for both *cis-* and *trans*-reaction profile is clearly less favorable and not competitive with the results above (Electronic Supplementary Material; Fig. S4). For the *cis*-reaction profile activation, barriers amount 30.0 and 27.8 kcal/mol for the direct and indirect pathways, respectively. For the *trans-*reaction profile, we were unable to obtain appropriate intermediates or transition states for the direct mechanism and the indirect mechanism has a barrier of 28.2 kcal/mol.

Totally, the reaction between ^**4**^**1**_**_Co_OS**_ and ethylene prefers the formation of interligand adducts with respect to the intraligand adduct, both thermodynamically and kinetically, thereby minimizing the decomposition route.

### Energy profile of 1__Co_OS_ for doublet spin state

**Fig. 3:**
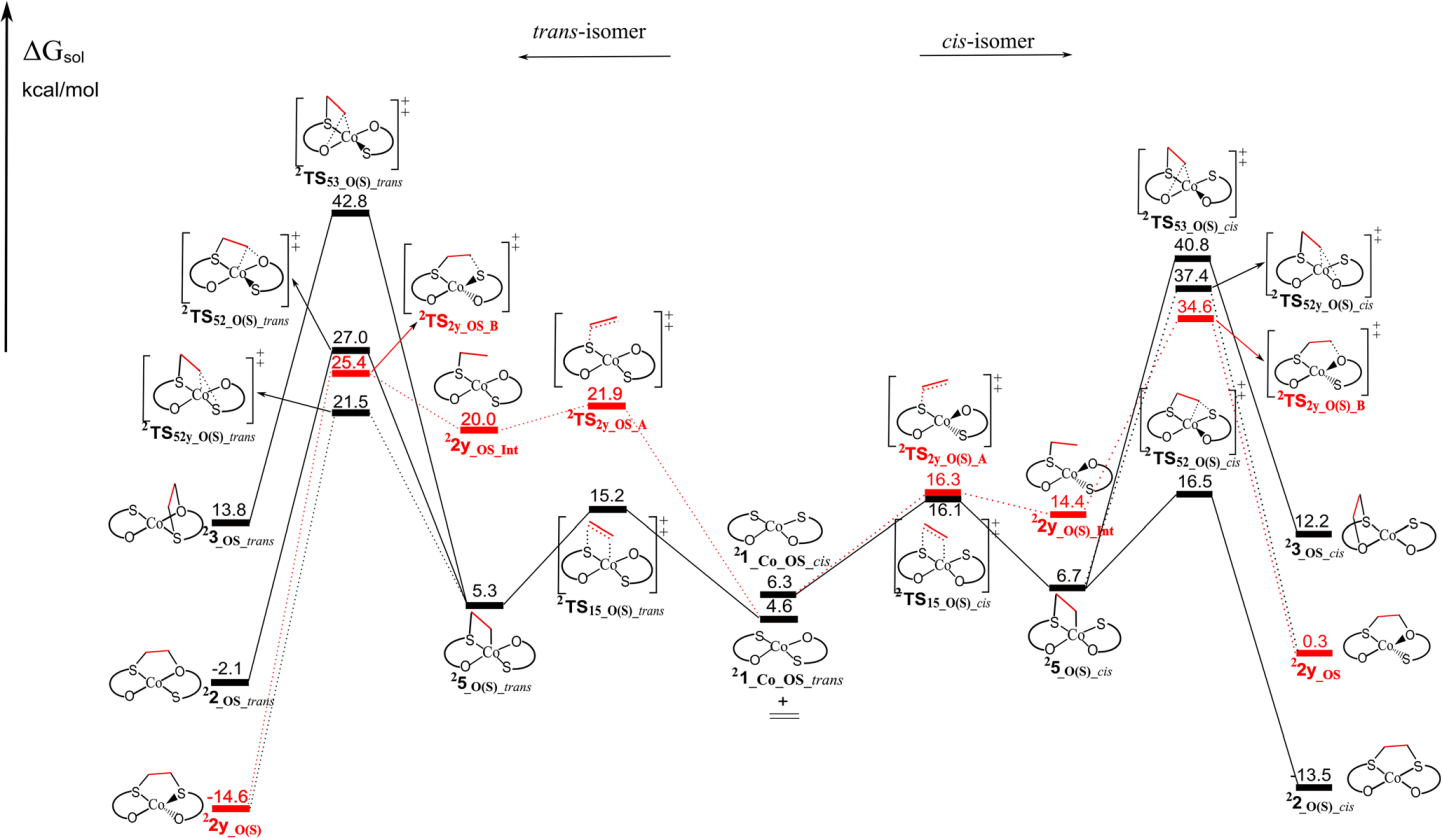
Calculated energy surfaces for the reaction of ^**2**^**1**_**_Co_OS**_ (*cis/trans*) with ethylene, along the S-binding side. Dotted (red) and solid (black) represent direct and indirect, respectively. Energies in kcal/mol are the free energy in solvent (CHCl_3_)

**Fig. 4:**
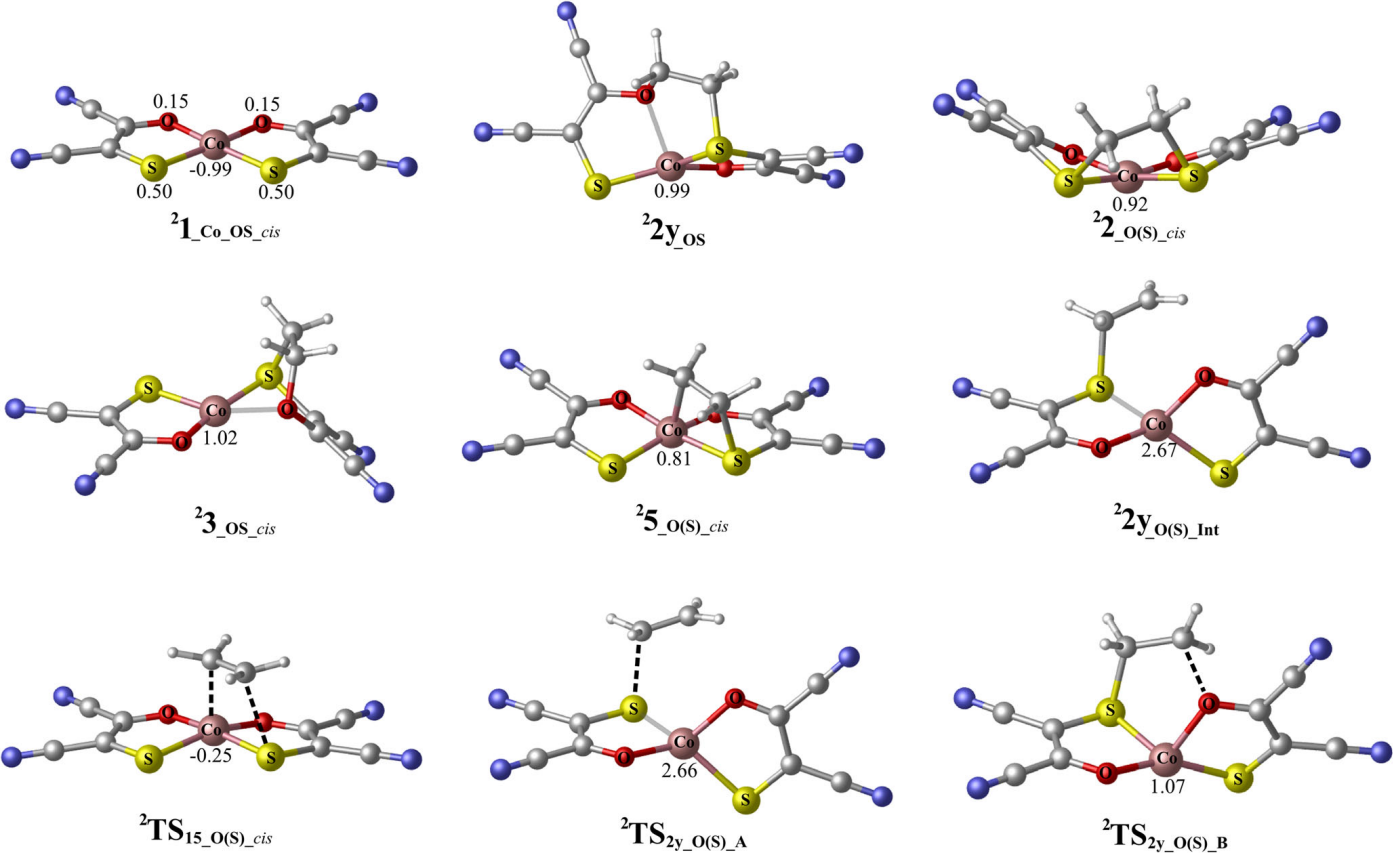
The optimized geometries for the selected species of *cis*-isomer that are included in Fig. 3. Values correspond to atomic spin densities. The geometries of selected species for *trans*-isomer are presented in Electronic Supplementary Material (Fig. S6)

On the other hand, doublet state energy profile for the reaction between ^**2**^**1**_**_Co_OS**_ (*cis/trans*) and ethylene is presented in Fig. 3 (optimized geometries of selected species in Fig. 4). This energy profile shows the direct (dotted lines) and indirect (solid lines) pathways that can act along the S-binding side (the energy profile for O-binding side is presented in Electronic Supplementary Material; Fig. S5). The *cis*-isomer is converted, through the direct mechanism, to generate ^**2**^**2y**_**_SO**_ (tetrahedral) in a stepwise mechanism involving the formation of intermediate ^**2**^**2y**_**_O(S)_Int**_ (with only one C-S bond a biradical electron distribution). This intermediate is formed with a barrier of 10.0 kcal/mol (^**2**^**TS**_**2y_O(S)_A**_) but needs to overcome the large additional barrier (formation of C–O bond) of 20.2 kcal/mol to further generate ^**2**^**2y**_**_SO**_ (^**2**^**TS**_**2y_O(S)_B**_). An appropriate transition state (or transition states) forming an intraligand adduct ^**2**^**3**_**_OS_*cis***_ in a direct pathway was not obtained, and this reaction is discarded. Regarding the indirect pathway, the intermediate ^**2**^**5**_**_O(S)_*cis***_ (only 0.4 kcal/mol relative to separate ^**2**^**1**_**_Co_OS*_cis***_ and ethylene), can be formed through the transition state ^**2**^**TS**_**15_O(S)_*cis***_ with a barrier of 9.8 kcal/mol (Fig. 3). **5**_**_O(S)_*cis***_ can isomerize into three different products: interligand adduct ^**2**^**2**_**_O(S)_*cis***_ via ^**2**^**TS**_**52_O(S)_*cis***_, interligand adduct ^**2**^**2y**_**_SO**_ via ^**2**^**TS**_**52y_O(S)_*cis***_, and intraligand adduct ^**2**^**3**_**_OS_*cis***_ through ^**2**^**TS**_**53_O(S)_*cis***_. The energy favored process is clearly the formation of ^**2**^**2**_**_O(S)_*cis***_, with a barrier of 9.8 kcal/mol; 24.3 kcal/mol more favorable than forming the intraligand adduct and 20.9 kcal/mol more favorable than forming ^**2**^**2y**_**_SO**_. Generally, the formation of desired interligand adduct ^**2**^**2**_**_O(S)_*cis***_ is both thermodynamically and kinetically driven. In respect to the O-binding side, the most favorable of the described pathways is the indirect pathway, but all the pathways must be considered as a noncompetitive due to the much higher energies (Electronic Supplementary Material; Fig. S5). By comparing the kinetic barriers, the preferred mechanism for the ethylene addition to ^**2**^**1**_**_Co_OS*_cis***_ is the formation of ^**2**^**2**_**_O(S)_*cis***_ by the indirect pathway along the S-binding side with a rate determining step of 9.8 kcal/mol. There is a significant kinetic preference for the formation of this product, with a barrier 24.3 more favorable than the formation of intraligand adduct ^**2**^**3**_**_OS_*cis***_. Furthermore, the intraligand adduct ^**2**^**3**_**_OS_*cis***_ is thermodynamically less stable than interligand adducts ^**2**^**2**_**_O(S)_*cis***_ and ^**2**^**2y**_**_SO**_ by 25.7 and 11.9 kcal/mol, respectively.

Figure 3 also shows the calculated energy profiles for the reaction of ^**2**^**1**_**_Co_OS*_trans***_ with ethylene. The direct pathway (dotted lines) was only found on the S-binding side, likewise previously described for the ^**4**^**1**_**_Co_OS**_. The direct pathway is also stepwise and it begins with the formation of intermediate ^**2**^**2y**_**_OS_Int**_ by one C–S bond (^**2**^**TS**_**2y_OS_A**_, 17.3 kcal/mol relative to separate ^**2**^**1**_**_Co_OS*_trans***_ and ethylene), followed by the formation of a second C–S bond producing the tetrahedral product ^**2**^**2y**_**_O(S)**_ with the barrier of 5.4 kcal/mol (^**2**^**TS**_**2y_OS_B**_). We could not locate the formation of the intraligand adduct ^**2**^**3**_**_OS_*trans***_ along the direct pathway. On the indirect pathway (solid lines), intermediate ^**2**^**5**_**_O(S)_*trans***_, 0.7 kcal/mol relative to the separate ^**2**^**1**_**_Co_OS*_trans***_ and ethylene, can be formed through the activation barrier of 10.6 kcal/mol (^**2**^**TS**_**15_O(S)_*trans***_). This intermediate can further isomerize into interligand adducts (^**2**^**2**_**_OS_*trans***_ (planar) and ^**2**^**2y**_**_O(S)**_ (tetrahedral)) or into intraligand adduct ^**2**^**3**_**_OS_*trans***_. Here, the barriers toward ^**2**^**2**_**_OS_*trans***_ (^**2**^**TS**_**52_O(S)_*trans***_) and ^**2**^**2y**_**_O(S)**_ (^**2**^**TS**_**52y_O(S)_*trans***_) are 15.8 and 21.3 kcal/mol lower in energy than one that leads to ^**2**^**3**_**_OS_*trans***_ (^**2**^**TS**_**53_O(S)_*trans***_), respectively (see Fig. 3). In addition, ^**2**^**2**_**_OS_*trans***_ and ^**2**^**2y**_**_O(S)**_ are more stable than ^**2**^**3**_**_OS_*trans***_ by 15.9 and 28.4 kcal/mol, respectively. The O-binding reaction was found through an indirect mechanism, but it has significantly higher energies and can be discarded without detailed discussion (Electronic Supplementary Material; Fig. S5). The energy preferred mechanism for the ethylene addition to ^**2**^**1**_**_Co_OS*_trans***_ is the indirect, S-binding route that leads to ^**4**^**2y**_**_O(S)**_ with a rate-determining barrier of 16.2 kcal/mol. On the other hand, the rate-determining barrier that leads to the formation of intraligand adduct ^**2**^**3**_**_OS_*trans***_ is 37.5 kcal/mol. Therefore, this pathway has kinetic selectivity toward the formation of the most stable interligand adduct of 21.3 kcal/mol. By comparing the two pathways, the direct pathway exclusively leads to ^**4**^**2y**_**_O(S)**_, with a rate-determining barrier 20.2 kcal/mol lower than the rate-determining barrier forming ^**2**^**3**_**_OS_*trans***_ through the indirect pathway. From the above, it can be concluded that the reaction between ^**2**^**1**_**_Co_OS_*trans***_ and ethylene prefers the formation of interligand adducts with respect to the intraligand adduct, both thermodynamically and kinetically, thereby minimizing the decomposition route.

In summary, all cobalt bis (oxothiolene) complexes (in both spin states) favor the formation of desired interligand adducts over the formation of the respective intraligand adducts, both kinetically and thermodynamically. This complex exceeds the performances of parent **1**_**_Ni_SS**_ complex [21, 22] for olefin purification and display better properties than **1**_**_Co_SS**_ [23]_**.**_ This can be viewed in terms of enhanced kinetic and thermodynamic selectivity toward the formation of interligand adduct, although the rate-determining barriers are somewhat lower in the case of **1**_**_Co_SS**_ (Electronic Supplementary Material; Fig. S7)_**.**_ The characteristics of **1**_**_Co_OS**_ complexes, as a potential catalyst, are quite similar with those for **1**_**_Ni_OS**_ complexes [24] that were previously published (Electronic Supplementary Material; Fig. S7). Thus, it can be concluded that oxothiolene complexes show better performances than dithiolene complexes as catalysts for the olefin purification process.

### The ethylene-release pathway from the reduced adducts

**Fig. 5:**
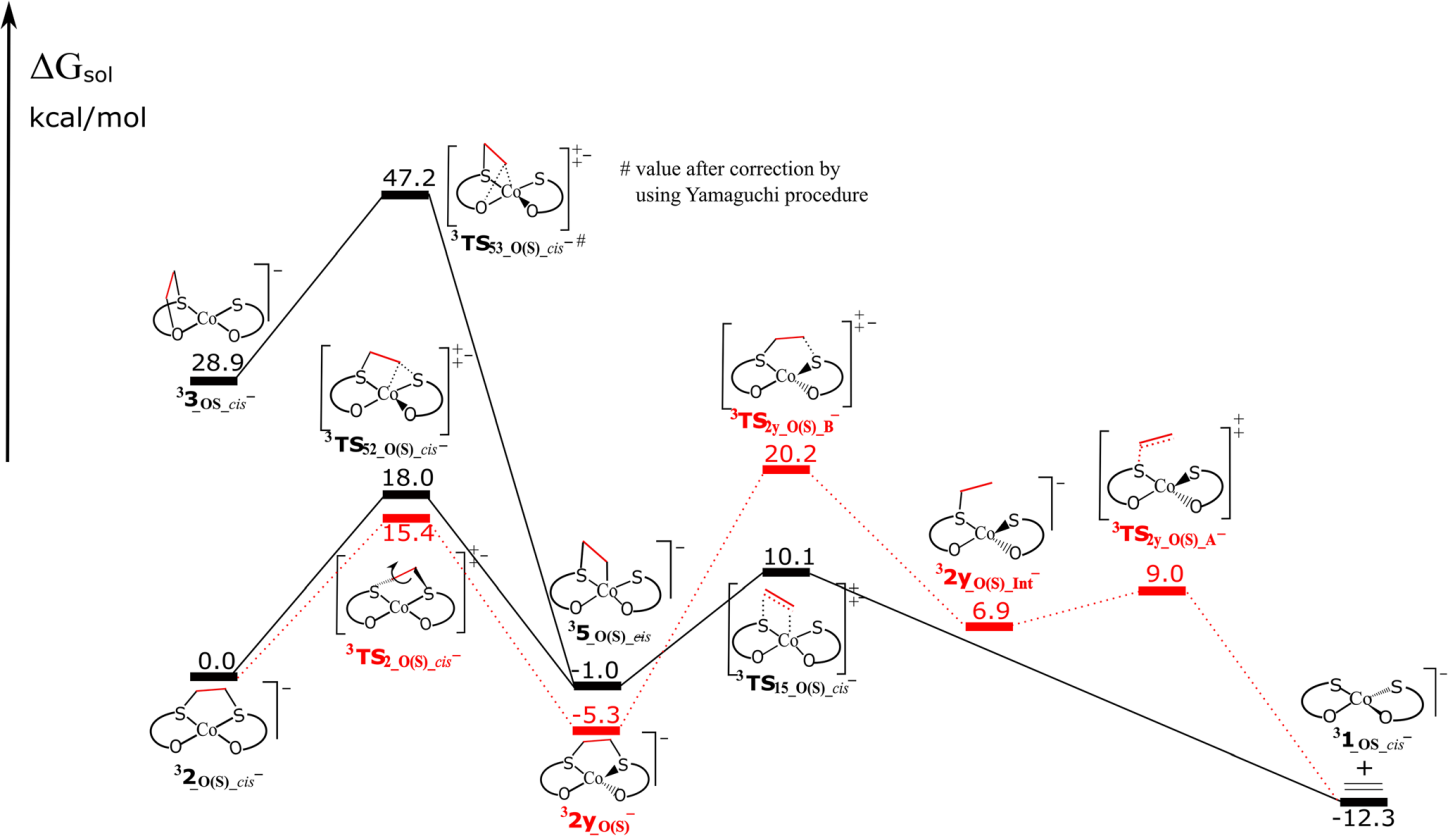
Calculated energy profiles for the release of ethylene from the anionic adducts ^**3**^**2**_**_O(S)_*cis***_^***−***^, ^**3**^**2y**_**_O(S)**_^**−**^, and ^**3**^**3**_**_OS_*cis***_^***−***^ via the direct (dotted lines) or the indirect pathway (solid lines). Energies in kcal/mol are the free energy in solvent

For being usable in electro-catalytic olefin purification, the complexes should release the olefin upon reduction. Considering that ^**3**^**2**_**_Co_OS**_^***−***^ and ^**3**^**2y**_**_Co_OS**_^***−***^ adducts are significantly more stable than their singlet counterparts (Table 1), we only present here the release of ethylene on the triplet state surface (Fig. 5), but an energy profile for the singlet state surface is presented in Electronic Supplementary Material (Fig. S8). The release of ethylene is favorable on both spin states, and even more favorable on the triplet state surface. Only the *cis* isomer ethylene releasing pathway from anionic adducts is shown, because *cis* neutral adduct is more stable than *trans* isomer. Therefore, following the indirect pathway ethylene can be released from ^**3**^**2**_**_O(S)_*cis***_^***−***^ by overcoming an activation barrier of 18.0 kcal/mol (^**3**^**TS**_**52_O(S)_*cis***_^***−***^), which is the rate-determining barrier for this pathway (Fig. 5). This process is exothermic by 12.3 kcal/mol. Alternatively, ^**3**^**2**_**_O(S)_*cis***_^***−***^ can isomerize into ^**3**^**2y**_**_O(S)**_^***−***^ by crossing a 15.4 kcal/mol barrier, from which ethylene can be released following the direct pathway with a rate-determining barrier of 25.5 kcal/mol (^**3**^**TS**_**2y_O(S)_B**_^***−***^). However, this is a more energy-demanding pathway, with a rate-determining barrier 7.5 kcal/mol higher than the indirect pathway. In addition, the release of ethylene from intraligand adduct ^**3**^**3**_**_OS_*cis***_^***−***^ is also possible through a barrier of 18.3 kcal/mol (^**3**^**TS**_**53_O(S)_*cis***_^***−***^), although the formation of this adduct is unfavorable. The rate-determining step for the ethylene release on the singlet state surface is 15.9 kcal/mol (corresponding also to the indirect mechanism; Fig. S8). Therefore, ethylene can be easily released from *cis*-interligand adduct **2**_**_O(S)_*cis***_^***−***^, on both states, following the indirect pathways, which are kinetically favored.

### Reaction mechanisms of copper bis (oxothiolene) complexes with ethylene

According to the thermodynamic data in Table 1, copper complexes (both neutral and cationic) might be alternative catalysts only if the intraligand adducts do not decompose. For the neutral complex in lower spin state (^**2**^**1**_**_Cu_OS_*cis***_), as well as for the cationic complex in higher spin state (^**3**^**1**_**_Cu_OS_*cis***_^***+***^), the formation of intraligand adduct **3** is slightly thermodynamically favored comparing to the formation of *cis*-interligand adduct **2**, whereas for the cationic complex in lower spin state (^**1**^**1**_**_Cu_OS**_^***+***^), the formation of adduct **3** is highly favored (Table 1). However, adducts ^**2**^**3**_**_Cu_OS_*cis***_ and ^**1**^**3**_**_Cu_OS_*cis***_^**+**^ have three-coordinated “broken” geometries that suggest that the decomposition process is accessible, whereas ^**3**^**3**_**_Cu_OS_*cis***_^**+**^ species represents the usual intraligand adduct (without breaking any metal-ligand bond). Even though ^**3**^**3**_**_Cu_OS_*cis***_^**+**^ does not favor the decomposition, it is 7.5 kcal/mol less stable than ^**1**^**3**_**_Cu_OS_*cis***_^**+**^ which does (Table 1). Therefore, neutral and cationic copper bis (oxothiolene) complexes can be excluded as alternative catalysts because of catalyst decomposition.

**Fig. 6:**
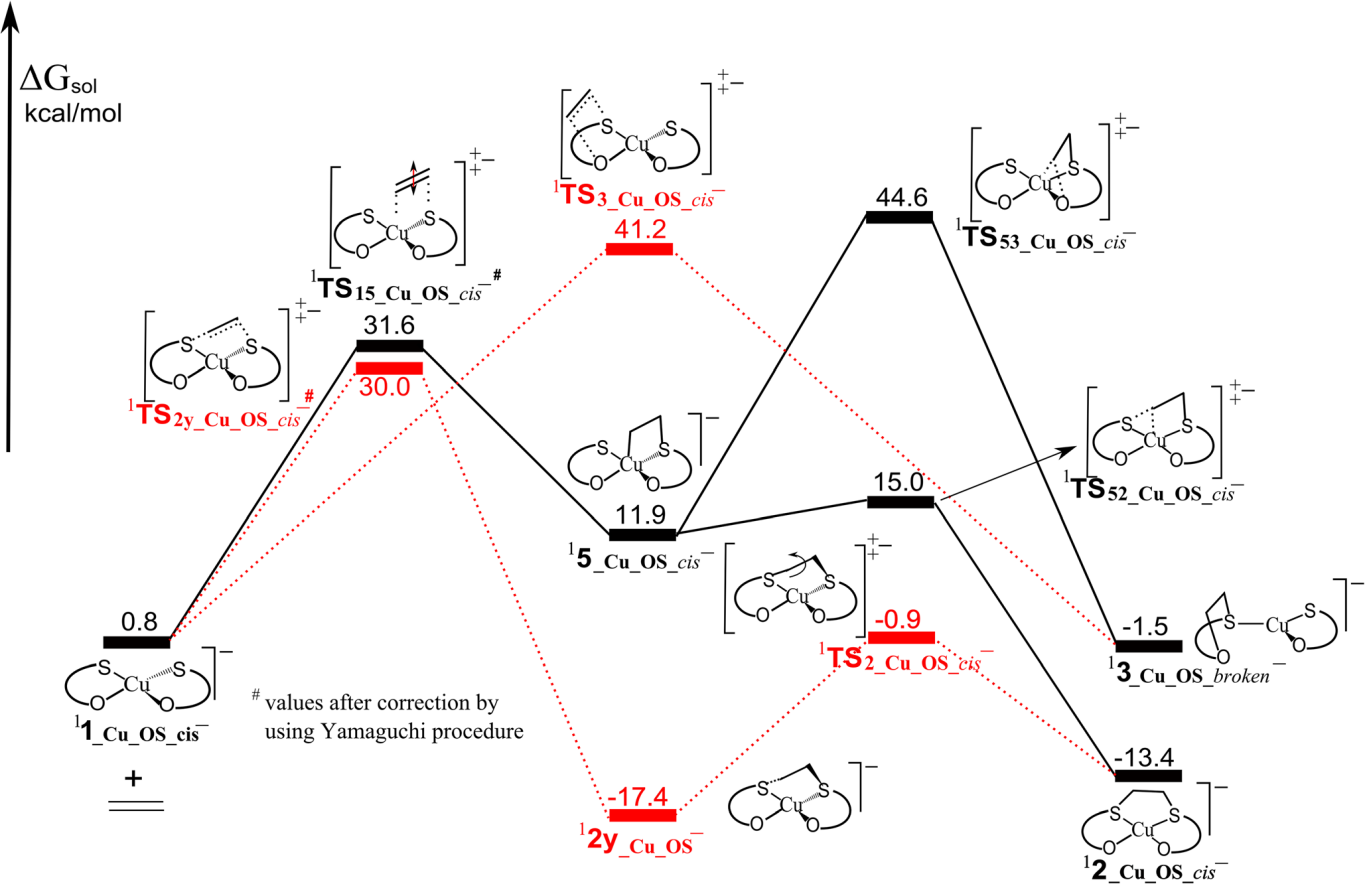
Calculated energy surfaces for the reaction of ^**1**^**1**_**_Cu_OS_*cis***_^***−***^ with ethylene. Dotted (red) and solid (black) lines represent direct and indirect pathway, respectively. Energies in kcal/mol are the free energy in solvent

On the other hand, the anionic copper complex ^**1**^**1**_**_Cu_OS_*cis***_^**−**^ might be a better alternative catalyst, because the reaction that leads to the formation of *cis*-interligand adducts **2** and **2y** are exothermic (− 13.4 and − 17.4 kcal/mol, respectively) and the reaction to form the intraligand adduct **3** is predicted to be close to zero free energy change. The complex in triplet state (^**3**^**1**_**_Cu_OS**_^**−**^), though more stable by 0.8 kcal/mol than the complex in singlet state (^**1**^**1**_**_Cu_OS_*cis***_^**−**^), exhibits unfavorable thermodynamics for the formation of all three adducts with ethylene (Table 1). Therefore, only the singlet-state energy surface for this complex was explored by locating all transition states (Fig. 6). Following the direct pathway, the twisted intermediate ^**1**^**2y**_**_Cu_OS**_^**−**^ (tetrahedral) is generated with an activation barrier of 28.2 kcal/mol (^**1**^**TS**_**2y_Cu_OS_*cis***_^**−**^). This product can further isomerize into ^**1**^**2**_**_Cu_OS_*cis***_^**−**^ (planar) through transition state ^**1**^**TS**_**2_Cu_OS_*cis***_^**−**^, by additionally passing the barrier of 16.5 kcal/mol. However, the second step is endothermic by 4.0 kcal/mol, indicating that the twisted, tetrahedral intermediate is the final product for this reaction. Alternatively, the intraligand adduct ^**1**^**3**_**_Cu_*broken***_^**−**^ can be formed directly with a higher barrier of 40.4 kcal/mol (^**1**^**TS**_**3_Cu_OS_*cis***_^**−**^). The indirect pathway begins with transition state ^**1**^**TS**_**15_Cu_OS_*cis***_^**−**^ imposing the energy barrier of 30.8 kcal/mol, which lead to the formation of intermediate ^**1**^**5**_**_Cu_OS_*cis***_^**−**^. This intermediate is 11.1 kcal/mol relative to the separate ^**1**^**1**_**_Cu_OS_*cis***_^**−**^ and ethylene and can subsequently isomerize into both interligand ^**1**^**2**_**_Cu_OS_*cis***_^**−**^ or intraligand ^**1**^**3**_**_Cu_*broken***_^**−**^ adducts, by overcoming the barriers of 3.1 kcal/mol (^**1**^**TS**_**52_Cu_OS_*cis***_^**−**^) and 32.7 kcal/mol (^**1**^**TS**_**53_Cu_OS_*cis***_^**−**^), respectively. Overall, the reaction of ^**1**^**1**_**_Cu_OS_*cis***_^**−**^ with ethylene favors the formation of the interligand adducts (^**1**^**2y**_**_Cu_OS**_^**−**^ and ^**1**^**2**_**_Cu_OS_*cis***_^**−**^) both kinetically and thermodynamically. Although the interligand adducts are enough thermodynamically stable, the barriers for their formation are somewhat higher that can be attributed to the fact that ethylene, which act as a nucleophile, approaches to the negative species. Therefore, ^**1**^**1**_**_Cu_OS_*cis***_^**−**^ cannot be considered as a good candidate for olefin separation process, but it shows somewhat better properties comparing to the previously published ^**1**^**1**_**_Cu_SS**_^**−**^complex [23]. Further modifications of ligands might lead to the copper complexes with enhanced catalytic properties. The relative stabilities of ^**2**^**2**_**_Cu_OS_*cis***_^**2−**^ and ^**2**^**2y**_**_Cu_OS_*cis***_^**2−**^ species indicate that the release of ethylene from adducts ^**1**^**2**_**_Cu_OS_*cis***_^**−**^ and ^**1**^**2y**_**_Cu_OS_*cis***_^**−**^ is thermodynamically favored (Table 1).

## Conclusion

In summary, we performed DFT calculations to predict a suitable catalyst for olefin purification. Inspired by our previous results, the differently charged cobalt and copper bis (oxothiolene) complexes of general formula [M (OSC_2_(CN)_2_)_2_]^*n*^ (M = Co, *n* = − 1, 0; M = Cu, *n* = + 1, 0, − 1, − 2) have been examined by exploring their reactivity with ethylene. For each complex, both the lower spin state (singlet or doublet) and the first higher spin state were considered to predict the thermodynamic stability of the *cis*-interligand and intraligand products. For those complexes with favorable thermodynamics (it is [Co (OSC_2_(CN)_2_)_2_] and [Cu (OSC_2_(CN)_2_)_2_]^**−**^), further mechanistic calculations were performed to explore kinetic feasibility of different pathways.

The results suggest that the neutral cobalt complex **1**_**_Co_OS**_ might be an alternative catalyst that performs better than the original **1**_**_Ni_SS**_. This is because **1**_**_Co_OS**_ preferably forms interligand adducts over the intraligand adducts, which are both kinetically and thermodynamically driven, thereby avoiding the decomposition issues of the nickel system. This happens with all the considered isomers and spin states, which includes two square-planar doublet species, *cis* and *trans*, and one tetrahedral complex in quartet state. In all cases, the direct pathways are stepwise and exclusively lead to the formation of interligand adducts. The indirect pathways also prefer formation of interligand adducts with relatively low-lying transition states, but intraligand products can be also formed with higher barriers. Consequently, **1**_**_Co_OS**_ complexes have no need for anionic species as a co-catalyst to produce desired interligand adduct, which are crucial for the original nickel complex. In addition, it was shown that upon reduction, the interligand adducts (^**1/3**^**2**_**_O(S)_*cis***_^***−***^ and ^**1/3**^**2y**_**_O(S)**_^**−**^) can easily release ethylene, following the indirect pathway, to produce ^**1/3**^**1**_**_Co_OS**_^**−**^ and regenerate the catalyst. It turns out that these complexes also perform better than analogous **1**_**_Co_SS**_ complex, by farther favoring the formation of the interligand adduct. According to the required performances for this type of catalysis, it appears that **1**_**_Co_OS**_ is comparable to the **1**_**_Ni_OS**_ complexes, thereby categorizing oxothiolene complexes as better candidates than the dithiolene complexes.

In the case of copper complexes, the neutral and cationic species are not good candidates for the catalyst due to the high relative thermodynamic stability of intraligand adducts, which have three-coordinated geometries (broken) that strongly indicate further decomposition. The mono-anionic complex **1**_**_Cu_OS_*cis***_^**−**^ might be considered as a potential catalyst, whose performances are somewhat better comparing to the analogous **1**_**_Cu_SS**_^**−**^ complex that was previously published. However, activation barriers for both direct and indirect pathways are somewhat higher and amount 30.0 and 31.6 kcal/mol, respectively. Therefore, further modifications of the ligand are required for obtaining the catalyst featured by lower activation barriers. Anyhow, all these results can give directive to the design of a series of novel complexes whose applicability can be validated experimentally.

## Electronic supplementary material

ESM 1 (DOCX 1146 kb)
