# Supplementary material for: Computational investigation of cobalt and copper bis (oxothiolene) complexes as an alternative for olefin purification
Source: J Mol Model. 2020 Jul 10;26(8):205. doi: 10.1007/s00894-020-04445-x (PMC7351838; doi:10.1007/s00894-020-04445-x)
Supplement: Supplementary file 1 — (DOCX 1146 kb) [file 894_2020_4445_MOESM1_ESM.docx]

Supporting Information

**Computational Investigation of Cobalt and Copper Bis(oxothiolene) Complexes as an Alternative for Olefin Purification**

Dušan N. Sredojević^1,2^ ‧ Rajesh K. Raju^1^ ‧ Salvador Moncho^1^ ‧ Milivoj R. Belić^1^ ‧ Edward N. Brothers^1^

^1^ Texas A&M University at Qatar, P.O.Box 23874 Doha, Science Department

^1^ Vinca Institute of Nuclear Sciences, P.O.Box 522, RS-11001 Belgrade, Serbia

E-mail: dusan.sredojevic@qatar.tamu.edu

dusredo@vinca.rs


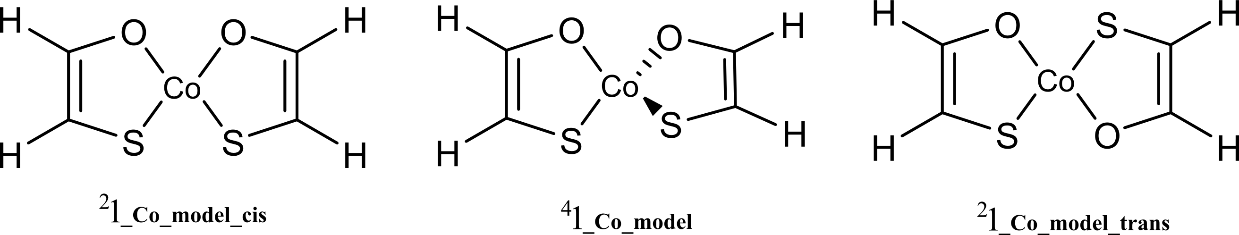


**Fig. S1** Three forms in two different spin states (doublet and quartet) of the cobalt model system **1__Co_model_**.

**Table S1**. Relative gas phase energies (in kcal/mol) for **1__Co_model_** at the ωB97X-D/6-31++G(d,p), CCSD(T)/6-31++G(d,p) and CCSD/6-31++G(d,p) level of theory.

|  | **^2^1__Co_model__*_cis_*** | **^4^1__Co_model_** | **^2^1__Co_model__*_trans_*** |
| --- | --- | --- | --- |
| ωB97X-D/6-31++G(d,p) | 5.0 | 0.0 | 8.2 |
| CCSD(T)/6-31++G(d,p) | 0.0 | 2.1 | 4.9 |
| CCSD/6-31++G(d,p) | 0.0 | 5.1 | 3.1 |


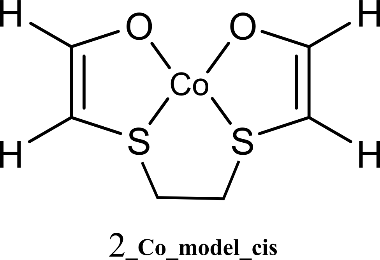


**Fig. S2**. The *cis*-interligand adduct of the cobalt model system **1__Co_model._**

**Table S2**. The relative gas phase energies (in kcal/mol) for **2__Co_model__*_cis_*** calculated at the ωB97X-D/6-31++G(d,p), CCSD(T)/6-31++G(d,p) and CCSD/6-31++G(d,p) level of theory.

|  | **^2^2__Co_model__*_cis_*** | **^4^2__Co_model__*_cis_*** |
| --- | --- | --- |
| ωB97X-D/6-31++G(d,p) | 6.5 | 0.0 |
| CCSD(T)/6-31++G(d,p) | 14.1 | 0.0 |
| CCSD/6-31++G(d,p) | 19.1 | 0.0 |


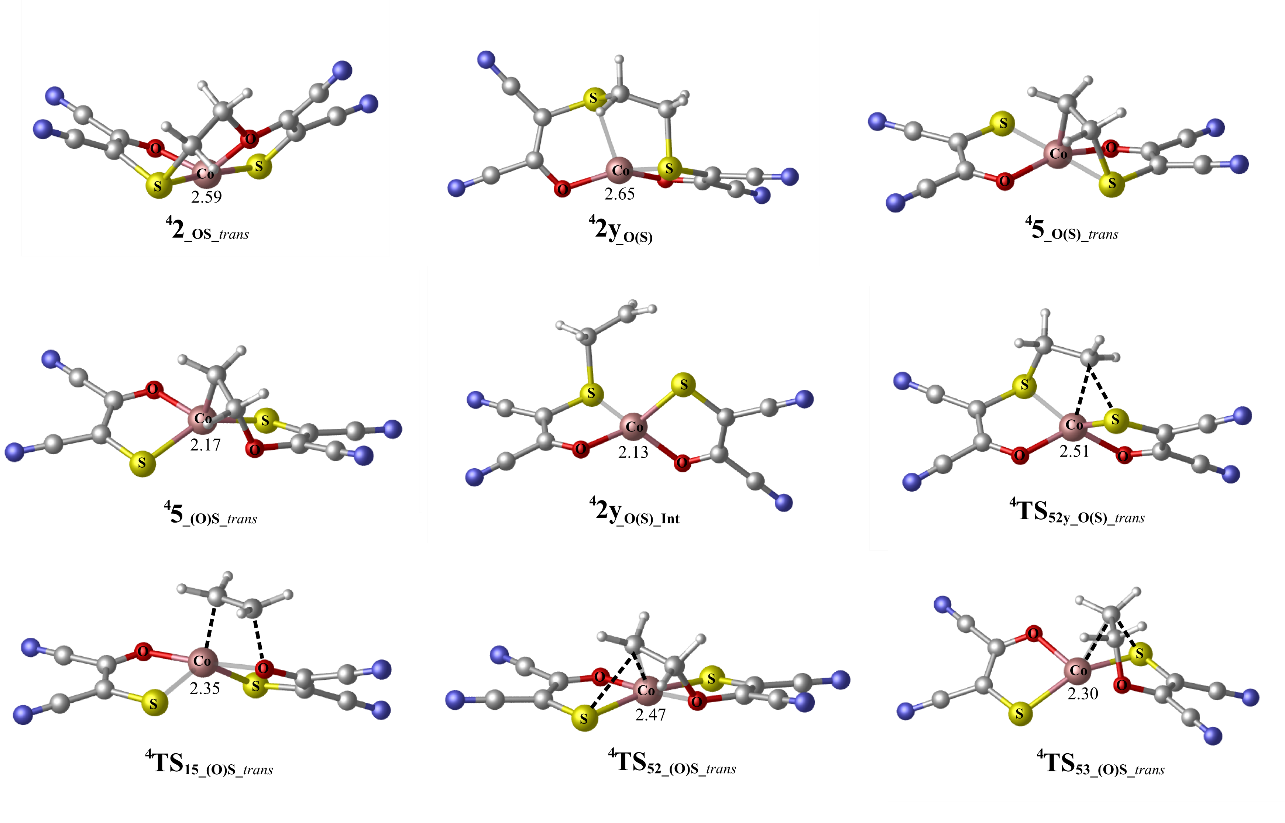


**Fig. S3** Optimized geometries for the selected species of *trans*-reaction profile that are included in Figure 1. Values correspond to atomic spin densities.


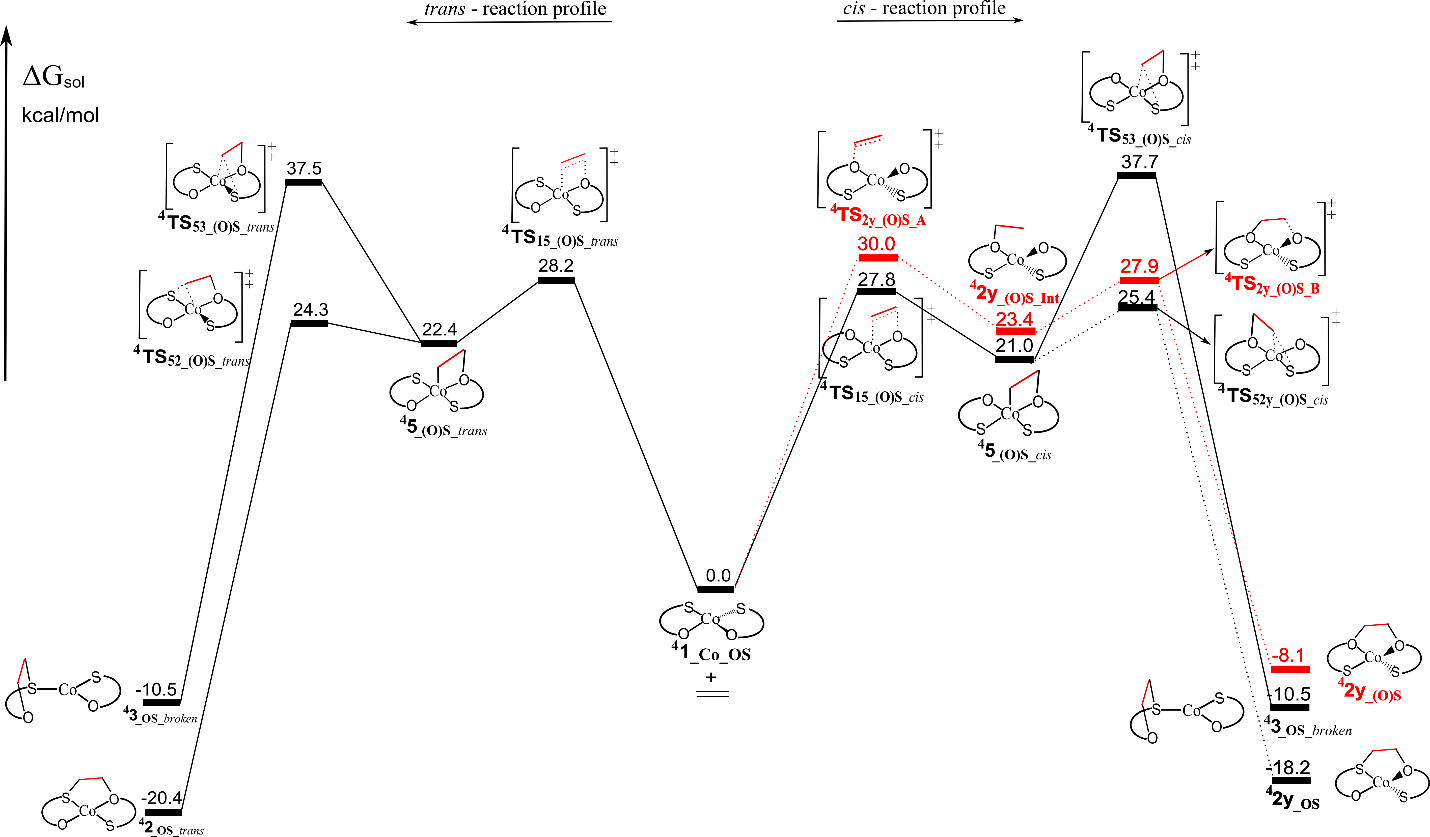


**Fig. S4** Calculated energy surfaces for the reaction of **^4^1__Co_OS_** with ethylene, for the *cis*- and *trans*-reaction profiles, considering O-binding side. Dotted (red) lines represent direct and solid (black) lines represent indirect pathway. Energies in kcal/mol are the free energy in solvent.


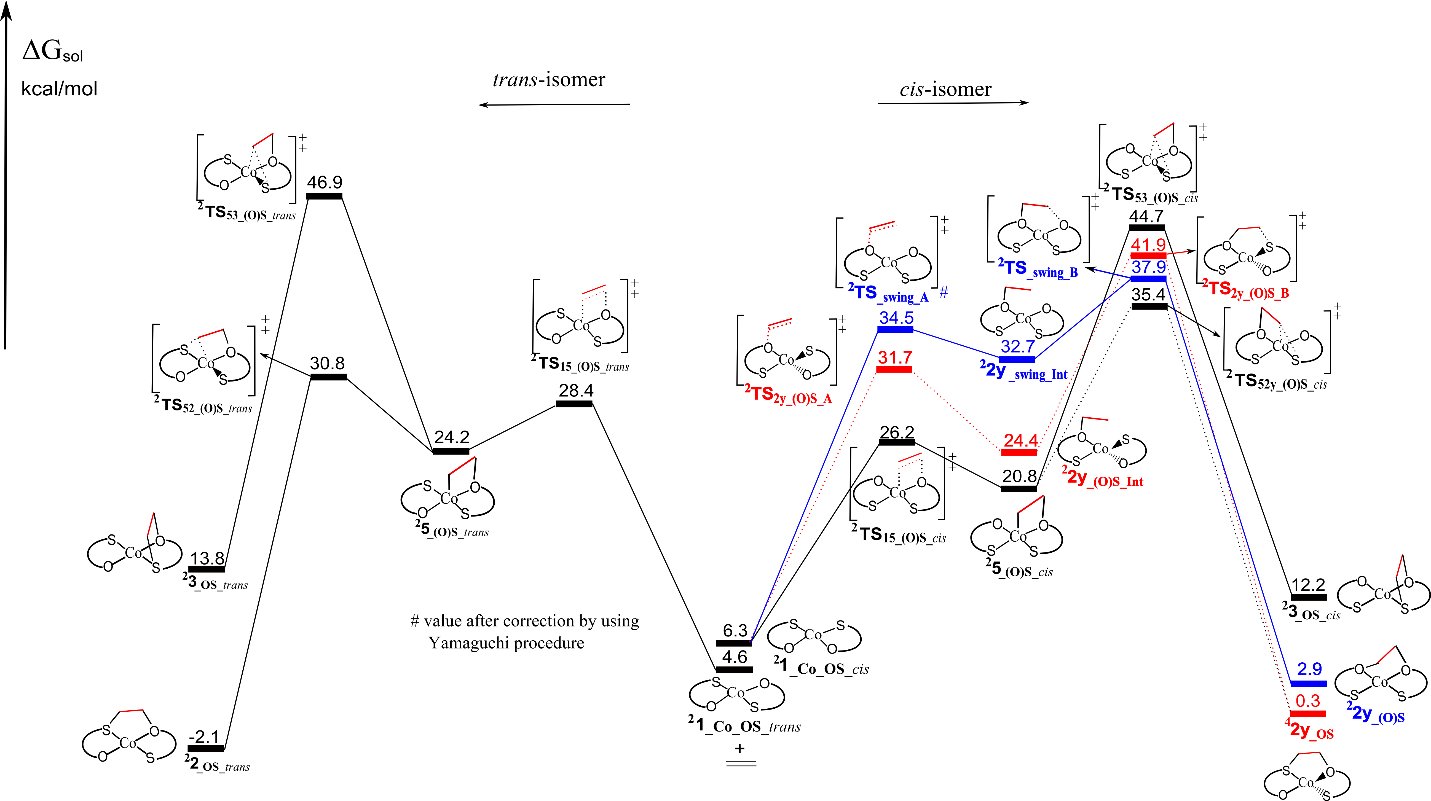


**Fig. S5** Calculated energy surfaces for the reaction of **^2^1__Co_OS_** (*cis/trans*) with ethylene, along the O-binding side. Dotted (red), solid (black), and solid (blue) represent direct, indirect, and swing mechanism respectively. Energies in kcal/mol are the free energy in solvent.


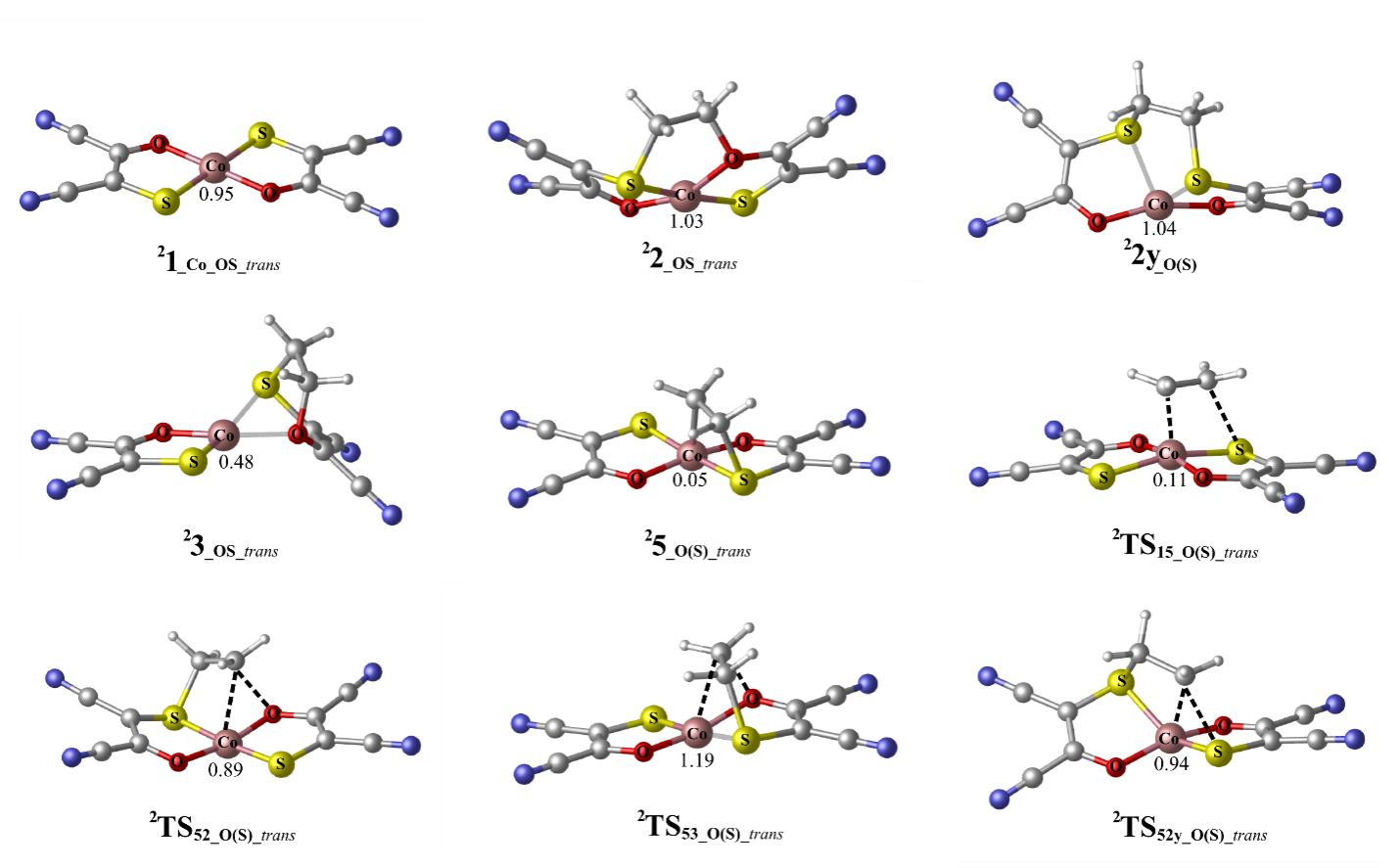


**Fig. S6** Optimized geometries for the selected species of *trans*-isomer that are included in Figure 3. Values correspond to atomic spin densities.


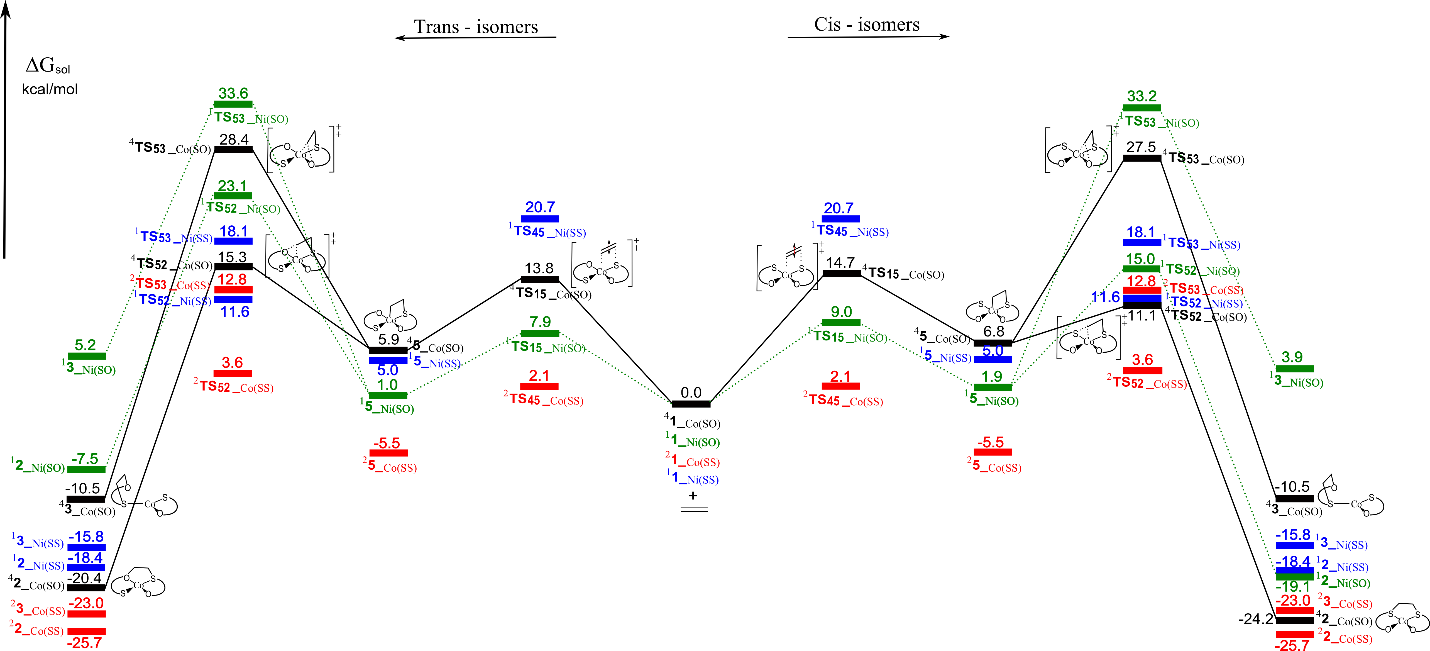


**Fig. S7** Comparisons of energy profiles of **^4^1__Co_OS_** (*cis/trans*) complex with those for previously published complexes (**^2^1__Co_SS,_ ^1^1__Ni_OS,_** and the original **^1^1__Ni_SS_**).


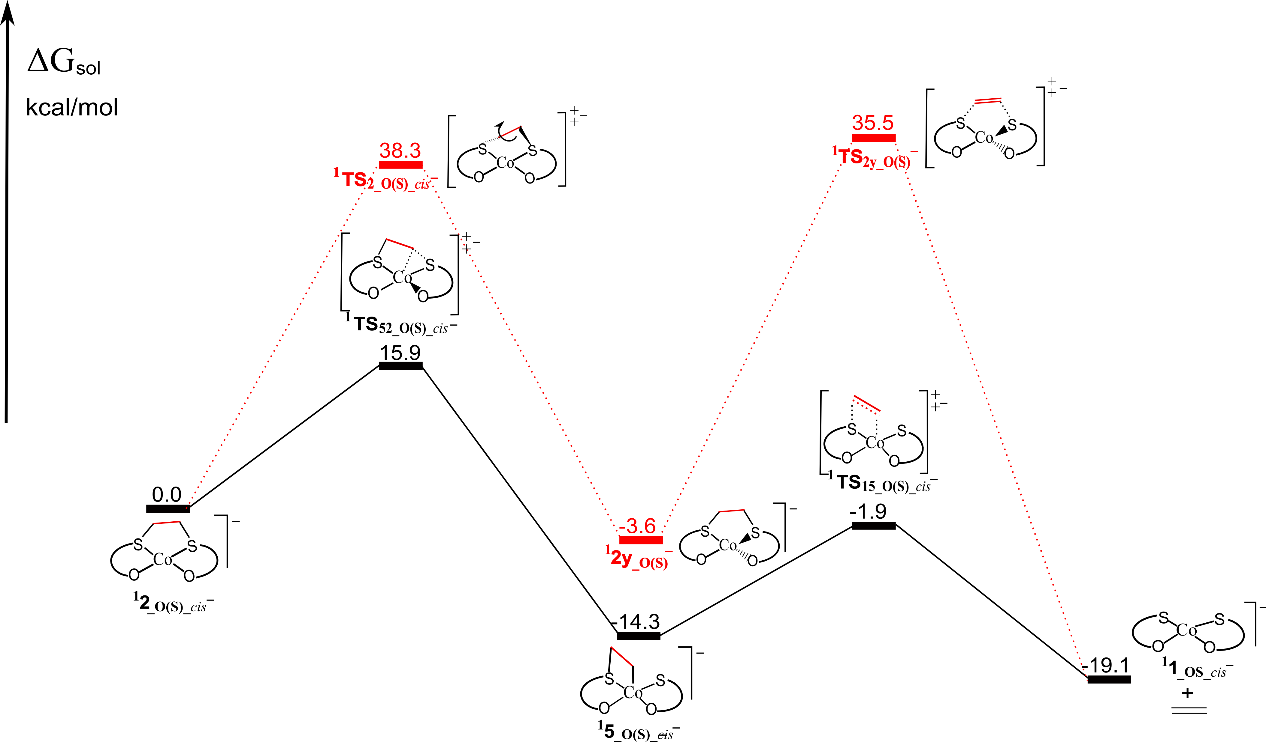


**Fig. S8** Calculated energy profiles for the release of ethylene from the anionic adducts **^1^2__O(S)__*_cis_^-^***, and **^1^2y__O(S)_^-^** via the direct (dotted lines) or the indirect pathway (solid lines). Energies in kcal/mol are the free energy in solvent.

**Table S3**. Calculated one electron reduction potentials with respect to Fc/Fc^+^.

| Half reactions | E^0^ (V) |
| --- | --- |
| Ni(mnt)_2_ + e^−^ → [Ni(mnt)_2_]^−^ | 0.69^a^ |
| **^1^1__Ni_SS_**+ e^−^ →**^2^1__Ni_SS_**^−^ | 0.76 |
| **^1^2__Ni_SS_**+ e^−^ →**^2^2__Ni_SS_**^−^ | -0.83 |
| **^2^1__Co_SS_**+ e^−^ →**^3^1__Co_SS_**^−^ | 1.15 |
| **^2^2__Co_SS_**+ e^−^ →**^3^2__Co_SS_**^−^ | -1.10 |
| **^4^2y__Co_SS_**+ e^−^ →**^3^2y__Co_SS_**^−^ | -0.93 |
| **^4^1__Co_OS_**+ e^−^ →**^3^1__Co_OS_**^− a^ | 0.57 |
| **^2^1__Co_OS__*_cis_***+ e^−^ →**^3^1__Co_OS_**^−a^ | 0.84 |
| **^2^1__Co_OS__*_trans_***+ e^−^ →**^3^1__Co_OS_**^−a^ | 0.77 |
| **^4^2__Co_O(S)__*_cis_***+ e^−^ →**^3^2__Co_O(S)__*_cis_***^−^ | -1.01 |
| **^4^2__Co_O(S)__*_trans_***+ e^−^ →**^3^2__Co_OS__*_trans_***^−^ | -0.94 |
| **^4^2y__Co_OS_**+ e^−^ →**^3^2y__Co_OS_**^−^ | -0.94 |
| **^4^2y__Co_(O)S_**+ e^−^ →**^3^2y__Co_(O)S_**^−^ | -0.94 |
| **^4^2y__Co_O(S)_**+ e^−^ →**^3^2y__Co_O(S)_**^−^ | -0.92 |
| **^1^1__Cu_SS_**^−^+ e^−^ →**^2^1__Cu_SS_^2^**^−^ | -1.00 |
| **^1^2y__Cu_SS_**^−^+ e^−^ →**^2^2y__Cu_SS_^2^**^−^ | -3.17 |
| **^3^1__Cu_OS_**^−^+ e^−^ →**^2^1__Cu_OS_^2^**^−^ | -0.72 |
| **^1^2y__Cu_OS_**^−^+ e^−^ →**^2^2y__Cu_OS_^2^**^−^ | -3.17 |

**^4^1__Co_OS_**

E = -2853.02171883 a.u.

Co 0.00059600 -0.06609500 -0.02455100

S 1.64899600 -1.18570100 1.21334400

C 2.90296800 -0.30176800 0.48017200

C 2.61886000 0.69121000 -0.50968400

S -1.66572000 -1.15091800 -1.22163200

C -2.92295800 -0.28125100 -0.48677900

C -2.61742200 0.70292900 0.50086300

C 3.71341400 1.42661500 -1.11101300

N 4.57817600 2.02073600 -1.60141900

C 4.25551800 -0.55078600 0.85389000

N 5.35306600 -0.76354400 1.16586900

C -4.27837800 -0.53462300 -0.84674000

N -5.37912600 -0.74804800 -1.14583600

C -3.68789800 1.44840200 1.13080800

N -4.53606100 2.04902900 1.64158100

O -1.43204800 0.95338200 0.85699700

O 1.44801700 0.96770000 -0.89495300

**^4^TS_15_O(S)__*_cis_***

E = -2931.57723368 a.u.

Co -0.05686400 -0.05507000 0.02666900

S 1.82127600 -1.53797000 -0.31440300

S -1.81505100 -1.49656200 -0.73422300

O 1.30797500 1.33840300 -0.21282900

O -1.55105100 1.27421600 0.31456200

N 5.50655800 -0.89995200 -0.21524100

N 4.23789600 3.00737500 -0.47356900

N -4.64209300 2.60074600 0.65122400

N -5.52413500 -1.04924100 -0.81771500

C 4.38340800 -0.61294500 -0.27689100

C 3.48913300 2.12692300 -0.40883700

C -4.41944000 -0.74514100 -0.63126300

C -3.80260200 1.84304100 0.40224500

C -3.05868800 -0.38898300 -0.40156400

C -2.73501200 0.90262300 0.10812600

C 2.99758100 -0.29172900 -0.33782500

C 2.53611300 1.03288900 -0.32460100

C 1.17458000 -1.64728000 1.89366900

C 0.07701200 -0.78851200 2.05776600

H 2.15584700 -1.33467400 2.23682500

H 1.00412300 -2.71961500 1.86733100

H 0.23863400 0.20477200 2.47723700

H -0.91835800 -1.20984400 2.16485600

**^4^5__O(S)__*_cis_***

E = -2931.59162967 a.u.

Co -0.08572400 0.11781900 -0.15921800

C 2.57436700 0.96327200 -0.40114900

S 1.76460500 -1.59314000 -0.20970500

C 2.99317300 -0.32981800 -0.17640500

C -2.78010300 0.89702300 0.15593000

C -3.04774500 -0.41504600 -0.34760000

C 4.34171000 -0.69148200 0.08366500

N 5.42902900 -1.02342300 0.32137700

C 3.55552500 2.03306700 -0.46981000

N 4.32382400 2.89708100 -0.52289800

C -3.88748600 1.74215000 0.56498400

N -4.76037500 2.42406000 0.90259700

C -4.39150000 -0.88009600 -0.43786400

N -5.48181400 -1.27197000 -0.51052200

C 1.20116400 -1.66830200 1.57384000

C 0.29014900 -0.50420600 1.76495200

H 0.74186000 0.42014700 2.12977700

H -0.70585500 -0.70269500 2.15896600

H 2.09306400 -1.65799800 2.20308700

H 0.68954900 -2.62763200 1.66891800

O 1.34190600 1.31276500 -0.54310500

S -1.76364200 -1.40950700 -0.84039400

O -1.61561500 1.35846000 0.26092600

**^4^TS_53_O(S)__*_cis_***

E = -2931.53688179 a.u.

Co -0.13289600 -0.00754300 -0.74339000

C 2.58867000 0.85755400 -0.18766900

S 1.72566700 -1.63288500 0.06194900

C 3.00454900 -0.44825400 -0.16979000

C -2.69138400 0.86535800 -0.15416700

C -3.00244800 -0.48259500 -0.29913100

C 4.36184800 -0.85869700 -0.25994400

N 5.46357700 -1.21540400 -0.33298300

C 3.52449000 1.95455300 -0.27611600

N 4.26674300 2.84047700 -0.34378700

C -3.68888400 1.81016700 0.30423700

N -4.46669700 2.58110000 0.68227500

C -4.29916100 -0.98845700 0.00029800

N -5.34761000 -1.42193600 0.24710800

C 1.08275300 -1.08429500 1.74686800

C 0.64138000 0.32277800 1.78757400

H 1.27462800 1.06879200 2.25108400

H -0.41617400 0.56443400 1.74002300

H 1.88929200 -1.28747900 2.45284600

H 0.23735700 -1.76437900 1.89810300

O 1.32760300 1.16906500 -0.04132600

S -1.76384600 -1.56608900 -0.85299000

O -1.51226400 1.32844600 -0.39057000

**^4^TS_52_O(S)__*_cis_***

E = -2931.58274618 a.u.

Co -0.09193500 0.14331200 -0.42626600

S -1.70092100 -1.45191600 -0.40100400

C -3.01194600 -0.42501300 -0.05981400

C -2.77667200 0.96829000 -0.16247400

S 1.84711800 -1.44262400 -0.74339800

C 2.98211100 -0.20673700 -0.17573200

C 2.51426600 1.07970200 -0.01611900

C -3.87447200 1.89960500 0.03385000

N -4.74469200 2.64734900 0.18701900

C -4.26131100 -0.95870300 0.36275000

N -5.27411400 -1.40863000 0.70765500

C 4.32752500 -0.59298800 0.06222300

N 5.41287200 -0.95247400 0.26780700

C 3.44760200 2.12829100 0.36561200

N 4.17412600 2.97400200 0.67633200

O 1.28979600 1.44455900 -0.17306800

O -1.63395600 1.43754100 -0.40722000

C 0.08744800 -1.16582600 1.36446700

C 1.14086700 -2.10178500 0.83544200

H 0.45989100 -0.24267900 1.82011200

H -0.68474500 -1.61939400 1.97931300

H 0.72551800 -3.08192800 0.58853300

H 1.96587400 -2.24031400 1.54103300

**^4^3__OS__*_broken_***

E = -2931.60023474 a.u.

Co 0.43564800 -1.09905800 -0.78910500

S -1.44433500 -1.52480100 0.57091700

C -2.63089300 -0.33459400 -0.02861300

C -2.78950900 0.89930700 0.51729800

S 2.30116500 -1.68482800 0.22360700

C 2.99679400 -0.07223000 -0.03087500

C 2.25253800 0.88264800 -0.66620800

C -3.85975400 1.75468000 0.07652900

N -4.72446200 2.43980500 -0.27311100

C -3.44415200 -0.79583200 -1.10085300

N -4.05960000 -1.22095000 -1.98750800

C 4.30980200 0.18077400 0.45681200

N 5.37777200 0.36414400 0.87380100

C 2.76115900 2.21412200 -0.89323200

N 3.13971000 3.29485900 -1.07168100

C -0.81428200 0.84745700 1.84007100

C -0.94332200 -0.64443500 2.09020300

H -1.68668400 -0.86267600 2.85965400

H 0.02380400 -1.06568800 2.37342000

H -0.09467600 1.06503600 1.04419600

H -0.51092400 1.35664900 2.75446500

O -2.07073400 1.44530200 1.49512200

O 1.02479300 0.65456300 -1.09457000

**^4^2__O(S)__*_cis_***

E = -2931.63391227 a.u.

Co 0.14117600 0.31960200 -1.14799600

C -2.14484600 1.20490000 0.24367600

S -1.85310600 -1.16369700 -1.01304400

C -2.75271900 0.00120700 -0.02365000

C 2.67674600 0.74440500 -0.33841300

S 1.36287600 -1.59448400 -0.28846100

C 2.57620200 -0.46049500 0.33008600

C -4.05112500 -0.35983100 0.41917300

N -5.09680100 -0.71111200 0.78338400

C -2.86314000 2.20940200 1.00972900

N -3.42129000 3.01515900 1.62515900

C 3.77997000 1.63397200 -0.01606800

N 4.65240100 2.35406300 0.22869100

C 3.46998300 -0.89969300 1.33961400

N 4.15763600 -1.29845200 2.18668000

C 0.05799000 -1.80380200 0.99488000

C -1.18346300 -2.31327100 0.26512200

H -0.12273700 -0.85436700 1.50585600

H 0.39588400 -2.54277500 1.72465100

H -1.99341700 -2.50454700 0.97311700

H -0.97675100 -3.24800900 -0.26435000

O 1.86868100 1.15001300 -1.26208400

O -0.95721300 1.52410900 -0.14186900

**^4^TS_2y_O(S)_A_**

E = -2931.57235876 a.u.

Co 0.10221500 -0.49893800 0.04254400

S -1.41341900 0.78754100 -1.21565200

C 3.04629700 -0.84027400 -0.14744400

C 2.70282500 0.24991200 0.70920900

C -2.79422800 0.06983800 -0.41985600

C -2.56763600 -0.83809500 0.60260500

C -1.25349100 2.67947100 -0.08393100

C -0.00594900 3.25780600 -0.33858400

H -1.41971700 2.24883300 0.90063800

H -2.13179000 3.13255500 -0.53632400

H 0.85167700 3.01340600 0.27893300

H 0.14672200 3.89848000 -1.20050400

C -4.09487000 0.48663900 -0.81450300

C -3.69503100 -1.44470400 1.28592200

C 4.42135000 -1.14492500 -0.37139200

C 3.75758800 1.02871500 1.32884100

N -5.13827400 0.86756600 -1.15335000

N -4.58324800 -1.93508400 1.84370800

N 4.57965300 1.67552600 1.82625800

N 5.53742300 -1.39893200 -0.56317300

O -1.39660300 -1.20060200 1.00041700

O 1.51042900 0.58896500 0.95637600

S 1.84173600 -1.77438300 -0.89770400

**^4^2y__O(S)__*_Int_***

E = -2931.57624256 a.u.

Co 0.06737200 -0.51153700 -0.03429100

S -1.49629600 0.98193800 -1.03220600

C 3.01443000 -0.63927600 -0.35911500

C 2.64274600 0.27108200 0.67679300

C -2.86700200 0.12451300 -0.30192300

C -2.59445300 -0.95981400 0.50133300

C -1.32458200 2.50518600 0.10614700

C -0.09943500 3.23742800 -0.22213700

H -1.33408700 2.11512700 1.12502900

H -2.23587000 3.07322800 -0.09143500

H 0.81892900 3.03092300 0.31489100

H -0.07983700 3.93742900 -1.04946500

C -4.17463600 0.60170300 -0.57788500

C -3.69584400 -1.70029300 1.09217400

C 4.39461800 -0.81770200 -0.66996300

C 3.67443000 0.99928800 1.38923000

N -5.22283100 1.04245600 -0.81534600

N -4.56105200 -2.29995700 1.57344300

N 4.47909900 1.60210400 1.96428400

N 5.51497600 -0.96761200 -0.93307600

O -1.40852100 -1.39556000 0.78023600

O 1.44293700 0.48935000 1.00947600

S 1.83651500 -1.51113600 -1.21929000

**^4^TS_2y_O(S)_B_**

E = -2931.57113754 a.u.

Co 0.03620500 -0.64292300 -0.22174800

S -1.51853700 0.86702600 -1.21255900

C 2.97151000 -0.58922100 -0.23717200

C 2.48603300 0.37901400 0.65887500

C -2.86804800 0.19690500 -0.27871900

C -2.59854500 -0.87224200 0.55164000

C -0.96740400 2.30754200 -0.17796900

C 0.49399400 2.51357900 -0.29589000

H -1.26144300 2.11658700 0.85783800

H -1.52699700 3.18165300 -0.53539800

H 0.98716600 3.12899900 0.44734600

H 0.99869400 2.37351900 -1.24745000

C -4.15636600 0.76361100 -0.45688700

C -3.69504200 -1.48027600 1.28873500

C 4.37678500 -0.81939400 -0.33936300

C 3.37883000 1.18772100 1.45276600

N -5.18881000 1.27133900 -0.61718500

N -4.55474500 -1.97400800 1.88591400

N 4.06057700 1.87996400 2.08443900

N 5.51717900 -1.01085600 -0.43346400

O -1.43589400 -1.39678800 0.74068500

O 1.22717600 0.62868800 0.76391700

S 1.90134300 -1.49112400 -1.22531500

**^4^2y__OS_**

E = -2931.63291251 a.u.

Co 0.00548000 -0.86994900 -0.33628400

S -1.48661500 0.84243400 -1.23014300

C 2.97597600 -0.55868000 -0.10367500

C 2.49222300 0.59225300 0.42123500

C -2.87085100 0.26681800 -0.28417300

C -2.65572400 -0.81593000 0.53911900

C -0.78747200 2.16316000 -0.14028700

C 0.72348800 2.11741000 -0.14089800

H -1.18281300 2.00274900 0.86503500

H -1.12664000 3.13314200 -0.50843800

H 1.12078500 2.89357600 0.51941800

H 1.14744800 2.24066700 -1.14335000

C -4.11470900 0.93334800 -0.42769900

C -3.76316200 -1.34427500 1.31872600

C 4.38099400 -0.82518600 -0.00251900

C 3.26600400 1.58689800 1.08776700

N -5.10996000 1.51766800 -0.55940200

N -4.63287900 -1.77375400 1.95013900

N 3.84616600 2.44108900 1.61702300

N 5.51466400 -1.05582800 0.06241100

O -1.52413100 -1.41642500 0.68012600

O 1.12623000 0.82121100 0.36895700

S 1.97848500 -1.75611600 -0.90375800

**^4^TS_15_O(S)__*_trans_***

E = -2931.57966478 a.u.

Co 0.03857400 0.15018700 0.07401700

S 1.74728400 1.78578000 0.22790100

C 3.03080100 0.71548500 -0.06545900

C 2.75478200 -0.65140900 -0.34561400

C -2.66288600 0.90078700 -0.28701500

S -1.58536100 -1.53839200 -0.44181300

C -2.92850400 -0.46965300 -0.40170200

C 3.84910000 -1.56087000 -0.63609300

N 4.70492900 -2.30773600 -0.86199400

C 4.37795700 1.18374900 -0.05694300

N 5.47090100 1.57383200 -0.04156900

C -3.75897800 1.85387400 -0.32007000

N -4.62122400 2.62606100 -0.34083900

C -4.25488500 -0.98747800 -0.39318900

N -5.32631300 -1.43397200 -0.37556200

O -1.49027600 1.36883000 -0.12387900

O 1.58456900 -1.12360400 -0.34681000

C -0.08308400 -0.65515700 2.07127600

H 0.97036000 -0.88188500 2.20695500

H -0.46215500 0.24186800 2.55929900

C -0.97488500 -1.69800400 1.77016600

H -2.00879800 -1.62649000 2.09258900

H -0.58057800 -2.70378800 1.65841700

**^4^5__O(S)__*_trans_***

E = -2931.59329477 a.u.

Co -0.07893400 0.28000000 -0.16681400

S -1.78890500 1.88132200 0.17412400

S 1.53894700 -1.58057400 -0.36595300

O 1.46358300 1.37436500 -0.43897700

O -1.59647300 -1.00603200 -0.50087800

C 2.64564300 0.87169000 -0.32289300

C 2.90744100 -0.47648800 -0.21309500

C -3.05899400 0.77124400 0.02014600

C -2.77399100 -0.57911800 -0.33151100

C -4.41014300 1.18304700 0.22079000

C -3.86137600 -1.52437600 -0.49842400

C 3.74565800 1.82085700 -0.29170800

C 4.20054600 -1.01569200 0.01830100

N -5.50536000 1.52633200 0.39156100

N -4.71285300 -2.29874900 -0.62807600

N 5.23828000 -1.49266300 0.22953600

N 4.60762100 2.59278500 -0.26178300

C 0.23889000 -0.48079400 1.72800300

C 0.95795200 -1.74725100 1.40677500

H -0.76509800 -0.56305800 2.14092800

H 0.83614500 0.33051300 2.14630800

H 0.29903200 -2.61671200 1.40455200

H 1.83610700 -1.94123700 2.02593300

**^4^TS_53_O(S)__*_trans_***

E = -2931.53885683 a.u.

Co -0.13839400 0.28761800 -0.68393300

C 2.60132100 0.77607600 0.18036700

S 1.54173900 -1.56512200 -0.44742300

C 2.91941100 -0.48817800 -0.23850800

S -1.79874800 1.81700500 -0.53567300

C -3.01161200 0.64155100 -0.15542200

C -2.67529100 -0.70793700 -0.15269400

C 4.24472300 -0.96620500 -0.41840600

N 5.31953800 -1.37548300 -0.57258000

C 3.61493300 1.75944700 0.48392000

N 4.41768000 2.55525700 0.73442300

C -4.32983200 1.07498000 0.17128300

N -5.39717900 1.44343300 0.44006900

C -3.65245400 -1.71841000 0.19172000

N -4.40155000 -2.55471000 0.47886700

C 0.89714100 -1.55956100 1.33528600

C 0.54303000 -0.21397700 1.82577100

H 1.19638800 0.29092800 2.52622100

H -0.49811500 0.09651200 1.83983100

H 1.68063500 -2.03003200 1.92982500

H 0.00816500 -2.19085900 1.25202900

O 1.35848200 1.12334300 0.39957900

O -1.48547300 -1.12691600 -0.43177000

**^4^TS_52_O(S)__*_trans_***

E = -2931.56342735 a.u.

Co 0.06319100 -0.52154500 -0.50953600

C -2.61751400 -0.96034700 0.12851000

S -1.59380700 1.27593700 -1.00687300

C -2.87769400 0.35146200 -0.20733300

S 1.88208300 -1.93633400 -0.35195200

C 3.00666800 -0.68555500 -0.02092700

C 2.61669600 0.65116400 -0.03286900

C -4.12441100 0.99335500 0.01309300

N -5.11547300 1.56942600 0.20140500

C -3.68787700 -1.76849900 0.69104700

N -4.52491400 -2.42489000 1.14725400

C 4.36616100 -1.02413900 0.26562900

N 5.46577800 -1.30891400 0.49927200

C 3.50718600 1.72706700 0.31231100

N 4.17509500 2.62700700 0.60930600

C -0.87209100 2.25315700 0.38602900

C 0.01922600 1.41837500 1.23332500

H -0.40233400 0.54071600 1.72464000

H 0.82971400 1.90196200 1.76706300

H -1.70849600 2.66358400 0.96423100

H -0.32467100 3.07772300 -0.07347300

O 1.38914400 0.98579000 -0.32303000

O -1.48092900 -1.54961000 -0.01413600

**^4^TS_52y_O(S)__*_trans_***

E = -2931.57929802 a.u.

Co -0.06386400 -0.00473500 -0.20074700

S -1.51903900 1.10576900 1.10813500

S 1.68630500 -1.63025500 -0.11511500

O 1.34949100 1.32895500 -0.39370800

O -1.59900900 -0.34672900 -1.38997800

C 2.56516900 0.91591900 -0.35677300

C 2.95497500 -0.39785000 -0.17800600

C -2.92524400 0.53406900 0.33235200

C -2.74907800 -0.11253200 -0.90725600

C -4.20111000 0.65515600 0.95323600

C -3.90223000 -0.57407300 -1.65774400

C 3.59177600 1.93839300 -0.49928200

C 4.30550400 -0.82110700 -0.08034400

N -5.23293800 0.75970500 1.47375800

N -4.81452400 -0.94872900 -2.26396100

N 5.39986600 -1.19858200 0.01704300

N 4.39093500 2.76857500 -0.60766900

C -0.39472700 -1.11037600 1.64358200

C 1.05701800 -1.53346500 1.64595500

H -1.09117600 -1.82407200 1.19363400

H -0.74418500 -0.78601900 2.61933600

H 1.16577900 -2.52643400 2.09551400

H 1.69811000 -0.83325500 2.18431300

**^4^2__OS__*_trans_***

E = -2931.63652005 a.u.

Co -0.05916700 -1.22350400 -0.34967400

C 2.56044500 -0.93846900 0.55961700

S 1.28517600 0.61090800 -1.25086100

C 2.65095500 0.21584100 -0.18513600

S -2.10705300 -1.60737900 -1.14201500

C -2.90559500 -0.28197700 -0.31595800

C -2.28715600 0.55490700 0.55133400

C 3.76593500 1.09295000 -0.17070700

N 4.63827400 1.86012600 -0.16941600

C 3.68487700 -1.33451000 1.39150500

N 4.56745400 -1.65842700 2.06661000

C -4.30349300 -0.09585100 -0.57133400

N -5.43112100 0.04239200 -0.80002400

C -2.92899600 1.62841600 1.23590600

N -3.39840100 2.53193600 1.79251700

C -0.08420500 1.46487900 1.03340100

C 0.42921600 1.93932000 -0.31075100

H 0.72426000 1.10545700 1.67212800

H -0.61734100 2.26329600 1.55569300

H 1.12828900 2.76950200 -0.18138300

H -0.38726400 2.27272900 -0.95837100

O -0.95914700 0.32338300 0.86808600

O 1.53506000 -1.72202400 0.57958900

**^4^TS_2y_OS_A_**

E = -2931.57078242 a.u.

Co -0.09756600 -0.37720300 -0.11524400

S 1.49884000 0.80601600 1.15289400

S -1.72226400 0.78927100 -1.36691400

C -2.75867700 -0.84740600 0.54679400

C -3.00764300 0.11524700 -0.48184200

C 2.82142000 -0.10586300 0.46788200

C 2.54076300 -1.03725800 -0.52038800

C 1.65995900 2.67502800 -0.02551500

C 0.55308500 3.48130300 0.25137900

H 1.71965700 2.20633000 -1.00479500

H 2.61867700 2.95894100 0.40105200

H -0.35477600 3.41063500 -0.33858100

H 0.54709600 4.14200100 1.11162700

C 4.14115000 0.18042600 0.91251900

C 3.62288400 -1.80055000 -1.11392900

C -3.88055700 -1.42278300 1.26369200

C -4.34796800 0.50113300 -0.77399100

N 5.20342500 0.46159100 1.28819900

N 4.47551500 -2.41311400 -1.60210400

N -5.43401000 0.82970500 -1.01894900

N -4.76659000 -1.88699300 1.84728900

O -1.60083400 -1.23080700 0.86846400

O 1.35645500 -1.28656500 -0.96158300

**^4^2y__OS__*_Int_***

E = -2931.57978468 a.u.

Co -0.10969200 -0.28764200 -0.10026400

S 1.53907800 0.95083100 1.06672800

S -1.72831000 0.83069000 -1.33556000

C -2.76246800 -0.80618700 0.55030900

C -3.02505700 0.10273200 -0.51846500

C 2.84723000 -0.03727200 0.38344200

C 2.50313900 -1.03815800 -0.49742000

C 1.68330600 2.54887400 0.05887000

C 0.57365500 3.45470400 0.38444400

H 1.70435400 2.24912100 -0.99027400

H 2.65765600 2.94821400 0.35105200

H -0.31437200 3.48794700 -0.23613100

H 0.59973200 4.05204400 1.28855100

C 4.17700700 0.25427700 0.78221900

C 3.54922900 -1.88077200 -1.05236700

C -3.86701200 -1.42167000 1.25834500

C -4.36751000 0.40526500 -0.88797900

N 5.24834500 0.54886700 1.12138900

N 4.37013800 -2.55718500 -1.50875200

N -5.45696900 0.66192500 -1.19470200

N -4.74276100 -1.91621500 1.83227700

O -1.58972700 -1.09399700 0.91684400

O 1.29905000 -1.30730900 -0.88460100

**^4^TS_2y_OS_B_**

E = -2931.57935225 a.u.

Co -0.09527500 -0.35452300 -0.08229800

S 1.51904600 0.95320100 1.04359200

S -1.69153900 0.84784000 -1.26511600

C -2.75284800 -0.81707800 0.57522800

C -3.00123400 0.13997300 -0.45088300

C 2.85817600 -0.01132200 0.39080200

C 2.53916000 -1.04244900 -0.46586200

C 1.57353900 2.49251500 -0.02729100

C 0.30773900 3.24452300 0.08183400

H 1.78934800 2.17256700 -1.04804700

H 2.43232600 3.05369200 0.35912000

H -0.21935600 3.56030600 -0.80990400

H -0.04571900 3.59494200 1.04566800

C 4.17922000 0.32021200 0.78708300

C 3.60767200 -1.87130000 -0.99915300

C -3.86629400 -1.42606100 1.27514100

C -4.33689800 0.51629900 -0.77607800

N 5.24332700 0.64387700 1.12238000

N 4.44640600 -2.53704400 -1.43845100

N -5.41951500 0.83365200 -1.04790300

N -4.74870200 -1.91658500 1.84228600

O -1.58433000 -1.15765800 0.91476600

O 1.34379200 -1.35049400 -0.84895700

**^4^2y__O(S)_**

E = -2931.64601175 a.u.

Co 0.00032600 -0.83481100 -0.00159400

S 1.27741300 0.81038000 1.17939700

S -1.27833600 0.81136000 -1.17930200

C -2.72276700 -0.85235200 0.39578100

C -2.78796300 0.24419600 -0.43971100

C 2.78747500 0.24435400 0.43965500

C 2.72349700 -0.85310700 -0.39470500

C 0.75450000 2.22861900 0.10829900

C -0.75547900 2.22854700 -0.10676700

H 1.30553200 2.15464200 -0.83270800

H 1.05427300 3.14932900 0.61287200

H -1.05550400 3.14972100 -0.61033900

H -1.30644700 2.15336700 0.83418400

C 3.99138800 0.91178900 0.78420700

C 3.95807200 -1.38143100 -0.95233300

C -3.95669400 -1.38125900 0.95430200

C -3.99259800 0.90974100 -0.78534900

N 4.94943400 1.50131100 1.07375200

N 4.93011000 -1.81141700 -1.41027600

N -4.95141300 1.49756300 -1.07580200

N -4.92819400 -1.81171900 1.41294200

O -1.64134900 -1.46369800 0.74317900

O 1.64284500 -1.46626900 -0.74156400

**^2^1__Co_OS__*_cis_***

E = -2853.01106958 a.u.

Co 0.00000000 -0.14357800 -0.00020100

S 1.66471100 -1.64437100 -0.00011200

C 2.92666200 -0.50448500 -0.00003900

C 2.55876000 0.86686000 0.00008000

S -1.66471100 -1.64437100 -0.00010200

C -2.92666200 -0.50448500 -0.00002300

C -2.55876000 0.86686000 0.00009300

C 3.57017100 1.90244600 0.00018900

N 4.37962100 2.73040400 0.00027800

C 4.28951600 -0.91021300 0.00000700

N 5.39948200 -1.24949800 0.00004700

C -4.28951600 -0.91021300 0.00003100

N -5.39948200 -1.24949800 0.00007700

C -3.57017100 1.90244600 0.00020800

N -4.37962100 2.73040400 0.00030300

O -1.34485800 1.21928100 0.00004400

O 1.34485800 1.21928100 0.00003700

**^2^TS_15_O(S)__*_cis_***

E = -2931.58445579 a.u.

Co -0.02809500 -0.08229900 -0.00679300

S 1.58905500 -1.51353200 -0.31883700

S -1.65263100 -1.55954700 -0.41354800

O 1.24600700 1.28389500 -0.15461400

O -1.37627800 1.25853100 0.03671300

N 5.33316700 -1.17363200 -0.42834700

N 4.22403500 2.90315900 -0.21974700

N -4.42027400 2.74670300 0.10736900

N -5.39113800 -1.21812800 -0.52443900

C 4.23614600 -0.79548000 -0.39738200

C 3.45408000 2.03903100 -0.23880500

C -4.29023100 -0.86530700 -0.42288600

C -3.60525700 1.92905900 0.02049200

C -2.93595900 -0.44784500 -0.29345300

C -2.58633100 0.90397600 -0.07791400

C 2.88433200 -0.36118400 -0.35312200

C 2.48606600 0.96093000 -0.25495000

C 0.89909300 -1.25252100 1.93038500

C -0.05883000 -0.22672800 2.00905900

H 1.93371700 -1.04441200 2.18247000

H 0.58483300 -2.28785200 2.02555400

H 0.26289700 0.76789200 2.30833500

H -1.08265800 -0.49397300 2.25602300

**^2^5__O(S)__*_cis_***

E = -2931.60178712 a.u.

Co -0.05652900 -0.03446100 -0.20740900

S 1.61185000 -1.51569400 -0.27812900

S -1.68869500 -1.49841100 -0.56870600

O 1.21499000 1.32710600 -0.29925300

O -1.39828900 1.28250100 0.05746300

N 5.33761600 -1.10395000 0.00555200

N 4.17385400 2.96838100 -0.22866900

N -4.44012000 2.70716100 0.52210900

N -5.42498400 -1.20904300 -0.31031900

C 4.24512600 -0.72314400 -0.09757300

C 3.41707700 2.09295800 -0.24080800

C -4.32184300 -0.84946100 -0.28308300

C -3.62504300 1.91486900 0.30249700

C -2.96521000 -0.42321500 -0.24709000

C -2.60825700 0.91875300 0.03447000

C 2.89661600 -0.30271300 -0.20541000

C 2.46119200 1.00058900 -0.25142600

C 1.06740300 -1.63876100 1.52483800

C 0.15871600 -0.45486600 1.66544900

H 1.97192500 -1.63578300 2.13468900

H 0.55659300 -2.60009800 1.60580500

H 0.62401800 0.43378100 2.09299000

H -0.80956200 -0.66651600 2.11835400

**^2^TS_53_O(S)__*_cis_***

E = -2931.53220890 a.u.

Co -0.15671800 -0.08131300 -0.26609500

C 2.47438300 0.90862000 -0.12685200

S 1.56114900 -1.56294600 -0.01815900

C 2.85907500 -0.38894000 -0.30174400

C -2.69567000 0.86533800 -0.16416700

C -3.04433700 -0.44920800 -0.20785800

C 4.18487800 -0.82431400 -0.55755300

N 5.26169900 -1.20475800 -0.76163500

C 3.40662400 2.00622900 -0.19213900

N 4.15653000 2.88691500 -0.23621400

C -3.66534700 1.92787900 -0.04794100

N -4.43300600 2.78935300 0.05569400

C -4.37197100 -0.94554800 -0.15575200

N -5.44795000 -1.38018500 -0.10757100

C 1.27000800 -1.17216300 1.79874300

C 0.76557400 0.22314700 1.91538100

H 1.41485900 0.97006000 2.35569500

H -0.29395900 0.38910400 2.08981000

H 2.20481900 -1.34709200 2.33244600

H 0.49371100 -1.88810400 2.08550800

O 1.23454700 1.21378500 0.19566800

S -1.67617300 -1.56600100 -0.35602500

O -1.43553100 1.23485300 -0.20873800

**^2^TS_52_O(S)__*_cis_***

E = -2931.58143689 a.u.

Co -0.06801900 0.06639600 -0.43457000

S -1.61318200 -1.41775600 -0.44753900

C -2.94451300 -0.38135800 -0.13268600

C -2.61647400 0.97395600 -0.09622400

S 1.64523400 -1.42132100 -0.58767700

C 2.88954300 -0.24128100 -0.14560200

C 2.45233900 1.06048400 -0.05840400

C -3.62419700 1.98369800 0.15665000

N -4.43220600 2.78785400 0.35690200

C -4.23287600 -0.90890700 0.14910300

N -5.27814300 -1.35894100 0.37703100

C 4.22221700 -0.68352300 0.04000000

N 5.29771500 -1.08976900 0.20711100

C 3.40298200 2.12453000 0.21580400

N 4.15432700 2.97539100 0.44092700

O 1.22332200 1.41381500 -0.20582000

O -1.41326600 1.36545400 -0.25483500

C -0.09525200 -1.08545000 1.44299400

C 1.01695100 -2.02252300 1.05681900

H 0.21625400 -0.12880000 1.86726000

H -0.91298400 -1.51879200 2.01082500

H 0.66944400 -3.04567500 0.90035600

H 1.85433500 -2.02785400 1.75912300

**^2^3__OS__*_cis_***

E = -2931.57562443 a.u.

Co 0.21473000 -0.19057600 0.01780200

S -1.57912000 -1.60633000 0.22164400

C -2.69522500 -0.38575800 -0.47422100

C -2.48619300 0.86195800 -0.00663100

S 1.78482800 -1.61889300 -0.16900700

C 3.09049000 -0.41565000 -0.24147500

C 2.68443500 0.87829900 -0.15748300

C -3.25560300 2.02046100 -0.33351100

N -3.88899400 2.95624000 -0.58782400

C -3.71477800 -0.75478700 -1.39206500

N -4.54188400 -1.07769500 -2.13706600

C 4.43892800 -0.83522300 -0.37910800

N 5.53353400 -1.20838600 -0.48784600

C 3.59774000 1.99355400 -0.19117100

N 4.31723900 2.90176700 -0.21189300

C -1.64604000 0.37803900 2.16192600

C -1.90950900 -1.12633100 1.97126900

H -2.93860700 -1.40647000 2.20102200

H -1.22876700 -1.73046200 2.57431100

H -0.71510400 0.57200500 2.69297300

H -2.47623900 0.87696600 2.66642100

O -1.44344600 1.03934500 0.86932100

O 1.40656600 1.17893400 -0.02311700

**^2^2__O(S)__*_cis_***

E = -2931.62458478 a.u.

Co 0.01536700 0.25447300 -0.82046500

C -2.39545200 1.12855700 0.00461400

S -1.63788000 -1.28965400 -0.80955000

C -2.74194700 -0.20622200 0.06202300

C 2.49865500 1.02512600 -0.05417200

S 1.51325500 -1.40154700 -0.51112900

C 2.76547100 -0.32071200 0.10996000

C -3.94563100 -0.73235800 0.58782000

N -4.89833800 -1.22531000 1.03500700

C -3.32498200 2.12576300 0.50895200

N -4.05911000 2.92242900 0.91576900

C 3.50427700 2.00283400 0.32851000

N 4.30075200 2.78426500 0.63511900

C 3.96379100 -0.87076900 0.62660700

N 4.92123600 -1.36441000 1.06180200

C 0.48808900 -1.86864100 0.96497900

C -0.85470600 -2.39218900 0.45744100

H 0.37050500 -0.98710500 1.60300900

H 1.01953100 -2.63853400 1.52954800

H -1.56503800 -2.51974400 1.27767300

H -0.74101200 -3.35566900 -0.04528800

O 1.40494800 1.49282900 -0.53825200

O -1.29270600 1.57246600 -0.48622400

**^2^TS_2y_O(S)_A_**

E = -2931.57773797 a.u.

Co 0.11612800 -0.44083800 -0.07915200

S -1.45441400 0.83041200 -1.25041100

C 3.05386700 -0.84798400 -0.14855500

C 2.70179200 0.23941800 0.70257800

C -2.79445400 0.08115800 -0.41267000

C -2.51438200 -0.83775100 0.58649400

C -1.27550400 2.70354900 -0.10017800

C -0.05093300 3.30864000 -0.40351700

H -1.38690000 2.25531600 0.88435800

H -2.18181600 3.15026200 -0.50093700

H 0.84038600 3.06314900 0.16394800

H 0.04875000 3.97032000 -1.25722200

C -4.11407300 0.48606500 -0.75161000

C -3.60578000 -1.47067100 1.30407600

C 4.42132200 -1.22409500 -0.29481800

C 3.73630200 0.97307200 1.40429000

N -5.17413500 0.85812600 -1.04579200

N -4.46483600 -1.98199700 1.88804300

N 4.54695500 1.57999700 1.96626400

N 5.53227500 -1.53460800 -0.42081600

O -1.32413900 -1.18775300 0.93363100

O 1.50480100 0.61259200 0.86399000

S 1.84244700 -1.67706500 -0.99896300

**^2^2y__O(S)__*_Int_***

E = -2931.58136593 a.u.

Co 0.08309400 -0.41998300 -0.14995700

S -1.58472100 1.00581200 -1.02585800

C 3.03366900 -0.60652400 -0.36902300

C 2.64636100 0.29022200 0.66949900

C -2.87668700 0.06286400 -0.25745000

C -2.51285700 -1.02146100 0.50989500

C -1.43242300 2.50725100 0.14797500

C -0.25477200 3.30100500 -0.20659200

H -1.38317700 2.08572400 1.15325000

H -2.37523600 3.03815500 0.00381000

H 0.70234300 3.09050600 0.25655700

H -0.30801700 4.04992100 -0.98821200

C -4.21702300 0.47871500 -0.46628100

C -3.54852100 -1.82772400 1.13336600

C 4.41512000 -0.86367600 -0.60953400

C 3.65904800 0.95312600 1.46645200

N -5.29525700 0.87037500 -0.64925400

N -4.36047400 -2.47916800 1.63943400

N 4.45348500 1.50024000 2.10738000

N 5.53748100 -1.07652000 -0.81330400

O -1.29612900 -1.40100200 0.72661500

O 1.43557000 0.55156900 0.92047400

S 1.84640100 -1.34809900 -1.32745000

**^2^TS_2y_O(S)_B_**

E = -2931.54422684 a.u.

Co 0.01135300 -0.67395300 -0.39948200

S -1.39577600 1.06655300 -0.96824500

S 1.80782700 -0.60358300 -1.72802000

O -1.43751200 -1.44821600 0.50509500

O 1.18934800 0.12210500 1.02585700

N -5.11295800 1.45153300 -0.55526400

N -4.58615300 -2.19143500 1.48211200

N 4.03782700 0.56260100 2.79651200

N 5.46959900 -0.45956300 -0.94596100

C -4.09718300 0.90280800 -0.42719700

C -3.71106800 -1.61592600 0.98970000

C 4.33241200 -0.40601200 -0.72191100

C 3.34529100 0.29083000 1.90811200

C 2.93205300 -0.34121700 -0.46102400

C 2.45176700 -0.01455700 0.81655600

C -2.83185000 0.28139500 -0.28009600

C -2.60284000 -0.90292200 0.38025900

C -0.89608800 2.18186700 0.44031300

C 0.57085000 2.27863200 0.58923400

H -1.36468500 1.78838100 1.34510500

H -1.34254400 3.15934100 0.21570600

H 0.96945400 2.56785000 1.55424200

H 1.20546300 2.41117400 -0.28187800

**^2^2__O(S)__*_cis_***

E = -2931.62458478 a.u.

Co 0.01536700 0.25447300 -0.82046500

C -2.39545200 1.12855700 0.00461400

S -1.63788000 -1.28965400 -0.80955000

C -2.74194700 -0.20622200 0.06202300

C 2.49865500 1.02512600 -0.05417200

S 1.51325500 -1.40154700 -0.51112900

C 2.76547100 -0.32071200 0.10996000

C -3.94563100 -0.73235800 0.58782000

N -4.89833800 -1.22531000 1.03500700

C -3.32498200 2.12576300 0.50895200

N -4.05911000 2.92242900 0.91576900

C 3.50427700 2.00283400 0.32851000

N 4.30075200 2.78426500 0.63511900

C 3.96379100 -0.87076900 0.62660700

N 4.92123600 -1.36441000 1.06180200

C 0.48808900 -1.86864100 0.96497900

C -0.85470600 -2.39218900 0.45744100

H 0.37050500 -0.98710500 1.60300900

H 1.01953100 -2.63853400 1.52954800

H -1.56503800 -2.51974400 1.27767300

H -0.74101200 -3.35566900 -0.04528800

O 1.40494800 1.49282900 -0.53825200

O -1.29270600 1.57246600 -0.48622400

**^2^1__Co_OS__*_trans_***

E = -2853.01506909 a.u.

Co -0.00202700 -0.00139600 -0.00030300

S 1.49896400 1.67038700 -0.00010700

C 2.86290200 0.66043200 -0.00007900

C 2.63056700 -0.73576300 -0.00003500

C -2.63235000 0.73473900 -0.00000300

S -1.49742200 -1.66571300 -0.00025500

C -2.86540600 -0.65857600 -0.00016100

C 3.72931500 -1.67562800 0.00003900

N 4.60842600 -2.42935400 0.00010000

C 4.18401100 1.19235300 0.00001300

N 5.25689900 1.63388800 0.00008800

C -3.72778300 1.67824700 0.00013300

N -4.60417300 2.43510100 0.00024300

C -4.18505500 -1.19401800 -0.00012800

N -5.25666800 -1.63854400 -0.00009900

O -1.44896900 1.20296600 -0.00004100

O 1.44990100 -1.20669500 -0.00012100

**^2^TS_15_O(S)__*_trans_***

E = -2931.58767761 a.u.

Co 0.02307100 0.04495400 0.02679400

S 1.48609400 1.69783800 -0.04178400

C 2.86903700 0.71555100 -0.17866900

C 2.65148900 -0.67345600 -0.24187900

C -2.57530500 0.82328800 -0.21324400

S -1.42478400 -1.53744100 -0.42428500

C -2.83171800 -0.52201400 -0.39101900

C 3.75457200 -1.60358600 -0.35219100

N 4.63126400 -2.35587900 -0.43160600

C 4.17801400 1.27474700 -0.23155100

N 5.24010100 1.73998900 -0.26975700

C -3.64343900 1.79846800 -0.15212700

N -4.49252000 2.58339700 -0.09676100

C -4.13463900 -1.08416600 -0.46930200

N -5.18942600 -1.56458000 -0.53060300

O -1.37241000 1.26919800 -0.07017100

O 1.47522900 -1.15914900 -0.19602300

C 0.07337600 -0.16006400 2.01291900

H 1.11601100 -0.31912800 2.27453400

H -0.38382400 0.74078400 2.41420800

C -0.74257700 -1.29681300 1.81088000

H -1.79157200 -1.25270300 2.08435000

H -0.28584700 -2.28168100 1.82834500

**^2^5__O(S)__*_trans_***

E = -2931.60503617 a.u.

Co -0.05017900 0.10544800 -0.21819600

S -1.53055600 1.72610400 0.09773800

S 1.46750100 -1.55526700 -0.38374600

O 1.34082100 1.32795100 -0.28798800

O -1.50025100 -1.09077300 -0.44390900

C 2.55041200 0.87244600 -0.23215800

C 2.85311100 -0.46671100 -0.22326200

C -2.90144800 0.72985900 -0.00576500

C -2.67723100 -0.63576400 -0.28653600

C -4.21653000 1.24709900 0.17450400

C -3.77764200 -1.56746600 -0.40499600

C 3.60544400 1.86643900 -0.17287900

C 4.15710600 -1.00976500 -0.10425800

N -5.28362000 1.67637600 0.32593300

N -4.65166400 -2.32193700 -0.49365900

N 5.20886500 -1.48961400 0.00707500

N 4.43949900 2.66708300 -0.12057500

C 0.15212300 -0.41902200 1.63251200

C 0.87951300 -1.71017800 1.40024300

H -0.83141700 -0.52722100 2.08967700

H 0.74350700 0.35836900 2.11668200

H 0.22620000 -2.58390700 1.40168500

H 1.76015600 -1.89013800 2.01872300

**^2^TS_53_O(S)__*_trans_***

E = -2931.53005517 a.u.

Co -0.15826200 0.09492600 -0.27083800

C 2.55690100 0.77776400 -0.09708800

S 1.44632600 -1.62348700 -0.11855700

C 2.83624000 -0.53823100 -0.30180300

S -1.60958100 1.71549200 -0.21818000

C -3.01182100 0.64927400 -0.15888000

C -2.74963300 -0.67959700 -0.22272400

C 4.13344000 -1.05841000 -0.55393700

N 5.18406800 -1.50612100 -0.75661500

C 3.56669800 1.80348100 -0.11018200

N 4.37868400 2.62861800 -0.11321100

C -4.32057300 1.19476500 -0.04247500

N -5.37757600 1.66301900 0.05906700

C -3.75372200 -1.70608500 -0.19363000

N -4.54532900 -2.55246200 -0.15783500

C 1.10296100 -1.27886400 1.69669100

C 0.81733800 0.17043300 1.89908800

H 1.58727700 0.78020000 2.35700600

H -0.19723400 0.48235800 2.13204400

H 1.96350100 -1.62588200 2.27062500

H 0.21335800 -1.88297800 1.89419400

O 1.33256200 1.17561600 0.22608900

O -1.48602000 -1.10403300 -0.30904700

**^2^TS_52_O(S)__*_trans_***

E = -2931.56035530 a.u.

Co 0.03337700 -0.31552300 -0.49368000

C -2.54704600 -0.95662500 -0.04051400

S -1.50076300 1.37510700 -0.81265400

C -2.80526200 0.39540800 -0.12423600

S 1.54312000 -1.86165800 -0.05811300

C 2.86784600 -0.77367300 0.02717400

C 2.62442200 0.57620600 -0.13355900

C -4.04583000 1.01089900 0.17600900

N -5.03103200 1.56701400 0.44048300

C -3.61203100 -1.86681100 0.34607200

N -4.45141100 -2.59802800 0.66229700

C 4.18519500 -1.25939900 0.29455500

N 5.24942300 -1.66422900 0.51237800

C 3.61033100 1.60563000 0.01897400

N 4.37575400 2.46568000 0.15695400

C -0.75767400 2.25636300 0.63454300

C 0.22658800 1.35578800 1.29609600

H -0.15557000 0.46217800 1.79065200

H 1.09809000 1.79453400 1.76845300

H -1.56964900 2.55223700 1.30742300

H -0.28161800 3.15100100 0.23141600

O 1.38680800 0.98310600 -0.35711800

O -1.40537700 -1.49708300 -0.29435300

**^2^3__OS__*_trans_***

E = -2931.57266964 a.u.

Co -0.20247700 -0.02399500 0.09647100

S 1.53794400 1.47059100 0.87629000

C 2.64750100 0.67015500 -0.28071300

C 2.48805100 -0.66666100 -0.35664900

C -2.74915500 0.77388000 -0.26800300

C -3.03338600 -0.55274100 -0.19537000

C 3.26744300 -1.57184200 -1.13741300

N 3.90889000 -2.30773400 -1.76089500

C 3.61669800 1.41297800 -1.00793700

N 4.40301600 2.03518300 -1.58909600

C -3.75677600 1.78769400 -0.45406800

N -4.55196200 2.61833000 -0.59884600

C -4.33807700 -1.10120500 -0.30527400

N -5.39446900 -1.57704000 -0.38694000

C 1.75265000 -1.12807100 1.85526100

C 1.93080600 0.34113500 2.27807200

H 2.94872900 0.55967000 2.60375800

H 1.23445000 0.61355600 3.07366500

H 0.87410200 -1.58892400 2.30485600

H 2.64180500 -1.72701600 2.06184300

O 1.48470400 -1.21796800 0.41339900

S -1.63584600 -1.61666200 0.05322200

O -1.50703500 1.21229700 -0.15565200

**^2^2__OS__*_trans_***

E = -2931.60771233 a.u.

Co -0.02967600 -0.57252700 -0.91065100

C 2.32838400 -1.01889400 0.38749600

S 1.65915200 0.97128500 -1.26636100

C 2.71837000 0.22420100 -0.05673500

S -1.84235900 -1.81999200 -0.71062100

C -2.80520800 -0.59696000 0.07655400

C -2.36089300 0.66370500 0.30238200

C 3.92157700 0.88335200 0.29648300

N 4.87513800 1.48425700 0.57852600

C 3.20760800 -1.77176500 1.26584000

N 3.89815300 -2.37395100 1.97285000

C -4.14142200 -0.93819700 0.46712000

N -5.21806400 -1.23246800 0.77811500

C -3.10391100 1.73155100 0.87405500

N -3.65551400 2.63649400 1.34798700

C -0.24380700 1.87366500 0.58079000

C 0.83730700 2.34796300 -0.36564400

H 0.15602100 1.31710100 1.43437100

H -0.83961100 2.71859400 0.93571300

H 1.60392300 2.91193400 0.17076500

H 0.41720400 2.98551400 -1.14747000

O -1.09599000 0.97897900 -0.18609400

O 1.21461800 -1.58993700 0.07528600

**^2^TS_2y_OS_A_**

E = -2931.56833200 a.u.

Co -0.11872900 0.21415600 -0.25424100

S -1.67637500 1.79182400 0.13186700

S 1.43688900 -1.36705700 -0.63728700

O 1.27062700 1.43846000 -0.01633700

O -1.53741900 -1.03708200 -0.41535800

C 2.48419600 1.00997700 -0.11896000

C 2.79838800 -0.30168400 -0.40996700

C -3.00375100 0.73908600 0.01628200

C -2.72909300 -0.62027900 -0.26051800

C -4.34000200 1.20202100 0.18988300

C -3.79647500 -1.59045600 -0.37286300

C 3.53065000 1.99248000 0.08472200

C 4.11942900 -0.81583600 -0.48047600

N -5.42495100 1.58652900 0.33514700

N -4.64281000 -2.37638600 -0.45822900

N 5.18284500 -1.28070900 -0.52211000

N 4.36062700 2.78085800 0.25723100

C 1.01345800 -1.40493500 2.28937900

C 1.29759100 -2.36547000 1.31392100

H -0.01320600 -1.15615600 2.54244300

H 1.80527200 -0.83280200 2.76071800

H 0.50513900 -3.05148100 1.02355600

H 2.29827500 -2.79055200 1.28537200

**^2^2y__OS__*_Int_***

E = -2931.57236484 a.u.

Co -0.13053200 0.22590700 -0.24376600

S -1.69908900 1.80207400 0.10961500

S 1.44766200 -1.36272300 -0.63429400

O 1.25164400 1.46211400 -0.03222300

O -1.54192600 -1.04085200 -0.36759200

C 2.47025000 1.03301100 -0.09748000

C 2.81033700 -0.27360900 -0.34538100

C -3.01834400 0.73671200 0.02402900

C -2.73557500 -0.62793900 -0.21782000

C -4.35745000 1.19390700 0.19111700

C -3.79676700 -1.60788800 -0.29847100

C 3.50565900 2.03057600 0.10181500

C 4.13029900 -0.78561100 -0.39376100

N -5.44492700 1.57355900 0.32998400

N -4.63741700 -2.40215700 -0.35953400

N 5.19057800 -1.26005300 -0.41505200

N 4.32458400 2.83102400 0.26972900

C 1.11091300 -1.54449100 2.14931200

C 1.36817500 -2.37537600 0.97236400

H 0.09435700 -1.37799000 2.49100500

H 1.91970300 -1.01300500 2.63740800

H 0.56985700 -3.08901700 0.75845000

H 2.33082800 -2.89155400 0.99797100

**^2^TS_2y_OS_B_**

E = -2931.56360231 a.u.

Co -0.06643600 0.22308500 -0.79800400

S -1.35010800 1.62396000 0.38852800

S 1.28644100 -1.54756800 -0.23743800

O 1.44531900 1.31442700 -0.75929400

O -1.62141000 -0.81278700 -1.10993100

C 2.56699800 0.79160900 -0.38755700

C 2.72849300 -0.53243200 -0.05906100

C -2.77841600 0.71193200 0.23653200

C -2.70793000 -0.45359400 -0.55724300

C -3.97734600 1.08944900 0.90557900

C -3.87228300 -1.28969900 -0.75932300

C 3.70353700 1.69427600 -0.34152400

C 3.94236300 -1.12846300 0.36407000

N -4.94735100 1.40283300 1.46004300

N -4.79849400 -1.96650800 -0.91716600

N 4.90657600 -1.65607600 0.74043200

N 4.60063200 2.42428200 -0.29919600

C 0.34896500 -0.46477500 2.18348400

C 0.70835100 -1.74025600 1.52621300

H -0.59978500 -0.36641100 2.69740500

H 1.10701200 0.29410800 2.34516100

H -0.13225400 -2.43427400 1.45878700

H 1.54027500 -2.25208800 2.02529400

**^2^2y__O(S)_**

E = -2931.62549221 a.u.

Co 0.00708200 -0.90631100 -0.00997800

S -1.21313700 0.67621500 -1.25368900

S 1.19526900 0.66597900 1.24183800

C 2.68652200 -0.70227900 -0.50873900

C 2.75281800 0.20013100 0.52450700

C -2.75914400 0.19678400 -0.52353000

C -2.67628500 -0.69873800 0.51568300

C -0.74426100 2.11344100 -0.18377700

C 0.73396800 2.11029300 0.17797700

H -1.38133100 2.07129800 0.70266600

H -0.98078000 3.02452600 -0.73774900

H 0.97478000 3.01758400 0.73628900

H 1.37089400 2.06872100 -0.70862300

C -3.96238200 0.76259400 -1.01489900

C -3.88149400 -1.13385500 1.19749900

C 3.90042300 -1.13236000 -1.17759000

C 3.94574900 0.78258400 1.02149900

N -4.92688600 1.25661500 -1.43306200

N -4.83533300 -1.48364400 1.75182400

N 4.90066700 1.29115000 1.44409000

N 4.86158500 -1.47767500 -1.72199900

O 1.58285700 -1.21378300 -0.95085800

O -1.56593300 -1.19665800 0.95194300

**^3^2__O(S)__*_cis_*^-^**

E = -2931.75300877 a.u.

Co 0.08406900 0.03942700 -1.31406200

C -2.05324600 1.23798000 0.22167400

S -1.86981800 -1.20822400 -0.91980900

C -2.72527100 0.03639500 0.00509300

C 2.61470300 0.74814300 -0.39071800

S 1.39532000 -1.64843100 -0.11738300

C 2.55392200 -0.41573800 0.38285300

C -4.03895000 -0.23151800 0.45286400

N -5.11156700 -0.50308100 0.81993200

C -2.77418300 2.29985100 0.94206400

N -3.30690900 3.15828400 1.50921200

C 3.72210800 1.67827000 -0.11957700

N 4.58234500 2.43431100 0.05565100

C 3.48543000 -0.72349700 1.40290500

N 4.21793700 -1.01364600 2.26171900

C 0.04172200 -1.68572100 1.12609900

C -1.19622500 -2.26080900 0.43853400

H -0.13559700 -0.67099500 1.49069800

H 0.33490400 -2.32677700 1.96239800

H -2.00317600 -2.40981800 1.16076400

H -0.97324200 -3.23039000 -0.02028200

O 1.83444000 1.07690800 -1.32714200

O -0.87862500 1.50418200 -0.14975700

**^3^3__OS__*_cis_*^-^**

E = -2931.70089842 a.u.

Co 0.17885300 -0.19507500 0.10634500

S -1.58227900 -1.60466200 0.24906600

C -2.64255500 -0.43404500 -0.53811700

C -2.42700800 0.88678100 -0.06337500

S 1.77314400 -1.63218500 -0.20712000

C 3.06033300 -0.41483100 -0.28300200

C 2.66031300 0.88195600 -0.12581100

C -3.17524400 2.03943300 -0.28303000

N -3.81004300 3.00854500 -0.46125400

C -3.65751700 -0.85957300 -1.39982200

N -4.50107800 -1.22865300 -2.12114800

C 4.40983900 -0.80449800 -0.48113300

N 5.50827000 -1.15443300 -0.64020700

C 3.60569400 1.97858300 -0.14866900

N 4.35003700 2.86841600 -0.15759100

C -1.67256500 0.40543700 2.13116000

C -1.95244500 -1.10797400 1.98609500

H -2.99738000 -1.35743800 2.17952500

H -1.30668700 -1.70248300 2.63677600

H -0.79403100 0.59633300 2.75045000

H -2.53671800 0.93932000 2.53561500

O -1.35010700 0.98896000 0.84822100

O 1.40815400 1.20309100 0.07129700

**^3^TS_52_O(S)__*_cis_*^-^**

E = -2931.72453530 a.u.

Co -0.13989100 0.13586100 -0.69641800

S -1.60888200 -1.42987200 -0.37671900

S 1.95599000 -1.49820300 -0.77038200

O 1.34054100 1.46459000 -0.34171900

O -1.67078800 1.49321600 -0.53988000

C -2.76473600 0.97827700 -0.16163400

C -2.94805800 -0.38753500 0.05478800

C 2.51062100 1.07200700 -0.05170200

C 2.99370800 -0.22951400 -0.10707400

C -4.12546900 -0.95091800 0.60386900

N -5.08174200 -1.44223300 1.05218700

C -3.88981500 1.88723300 0.06400200

N -4.76213900 2.63215200 0.23058700

C 3.44118300 2.12165300 0.38884900

N 4.14380900 2.97602300 0.73271100

C 4.30985700 -0.57706300 0.28954400

N 5.37784600 -0.90455000 0.61928000

C 1.08157900 -2.12365000 0.71871900

C -0.01909400 -1.17763300 1.14799600

H 1.81853300 -2.28804600 1.51188500

H 0.67280900 -3.09638200 0.42683100

H 0.35429600 -0.21841800 1.52199500

H -0.66990900 -1.60535100 1.90933700

**^3^5__O(S)__*_cis_*^-^**

E = -2931.75697359 a.u.

Co -0.26404400 0.12773100 0.07856000

C 2.46489800 0.92352300 -0.30675200

S 2.01319100 -1.77657500 -0.30684600

C 3.02185800 -0.33127800 -0.16574200

C -2.86941600 1.02681600 0.00086000

C -3.15034700 -0.31217300 -0.15092600

C 4.41603300 -0.50579500 0.05752900

N 5.54914900 -0.68852200 0.24841400

C 3.36080900 2.08215000 -0.28814400

N 4.04296500 3.01825800 -0.27812400

C -3.94389300 2.00206200 0.07524000

N -4.78359100 2.79964100 0.13782200

C -4.47168900 -0.83130500 -0.16028000

N -5.54397500 -1.28297800 -0.17248900

C 1.27656900 -1.89345100 1.38093300

C 0.46296800 -0.67096200 1.72666500

H 1.07756200 0.16508900 2.08177600

H -0.34858500 -0.87585500 2.42625800

H 2.08684700 -2.07862200 2.09399200

H 0.64295900 -2.78369000 1.32902000

O 1.22415500 1.21135600 -0.45019700

S -1.78287700 -1.41639800 -0.28629300

O -1.66147800 1.48150700 0.09907100

**^3^TS_15_O(S)__*_cis_*^-^**

E = -2931.73078631 a.u.

Co -0.14636000 -0.00261800 0.04748300

C 2.52628700 0.96345400 -0.46535800

S 1.84146800 -1.61159700 -0.17873400

C 2.99768800 -0.34449600 -0.28784500

C -2.77890900 0.83554000 0.15236600

C -3.02988100 -0.37058300 -0.45898500

C 4.38587000 -0.62183000 -0.13246200

N 5.51149500 -0.87837100 0.00703800

C 3.49506600 2.04540100 -0.63696500

N 4.24446700 2.91815600 -0.77360000

C -3.87259100 1.71276500 0.52937300

N -4.72171400 2.43570300 0.85085900

C -4.35199400 -0.79958500 -0.75797900

N -5.42368300 -1.17689600 -1.01059900

C 1.13218100 -1.42994000 2.05641500

C 0.11553500 -0.48270100 2.16445300

H 0.34605700 0.53599500 2.46893600

H -0.91708400 -0.80401000 2.26757300

H 2.14384300 -1.17474800 2.35414500

H 0.87577300 -2.48448300 2.08884100

O 1.30325300 1.29080000 -0.47313800

S -1.66898900 -1.42117900 -0.86759600

O -1.58400300 1.25720800 0.44636100

**^3^1__OS__*_cis_*^-^**

E = -2853.18641983 a.u.

Co -0.06801700 -0.05736100 0.03371000

S 1.64207800 -1.16528200 1.20016600

C 2.91148200 -0.30711700 0.46810100

C 2.60668500 0.71779600 -0.46986800

S -1.59894100 -1.23290400 -1.12222400

C -2.92569900 -0.28084900 -0.43491400

C -2.64160200 0.69581600 0.48393500

C 3.69481500 1.47338700 -1.07991400

N 4.54516900 2.08532200 -1.57422500

C 4.26922400 -0.61484600 0.78038800

N 5.36956300 -0.87612500 1.04569600

C -4.25705100 -0.56359700 -0.84706200

N -5.33593100 -0.81646500 -1.20156800

C -3.69709300 1.49234200 1.07538300

N -4.52073800 2.14494900 1.56778100

O -1.42598200 0.97169700 0.88503500

O 1.43203900 1.01050400 -0.80950100

**^3^TS_53_O(S)__*_cis_*^-^**

E = -2931.65747243 a.u.

Co -0.17768300 -0.13623900 -0.35647300

C 2.50939300 0.88708500 -0.02507300

S 1.78279400 -1.65930400 0.00382800

C 2.97928500 -0.39441300 -0.24428700

C -2.73244200 0.91692500 -0.30915500

C -3.08644900 -0.39547000 -0.09864000

C 4.30237100 -0.70824900 -0.63545400

N 5.38557100 -0.98887000 -0.95449800

C 3.37176400 2.04068600 -0.05138900

N 4.06488900 2.97041400 -0.07229300

C -3.75767000 1.95114600 -0.33261100

N -4.54375500 2.80490400 -0.35223700

C -4.42834000 -0.80395500 0.10764200

N -5.51937900 -1.17196400 0.28653500

C 1.31290400 -1.27515300 1.74637400

C 0.80229700 0.13243800 1.87588700

H 1.42176600 0.82068400 2.44183900

H -0.26271600 0.26218500 2.04948200

H 2.18273600 -1.46477100 2.38151500

H 0.50489600 -1.98464000 1.95349300

O 1.26415400 1.10382200 0.31637300

S -1.79438400 -1.62053400 -0.12425200

O -1.51480700 1.31798300 -0.48610300

**^3^TS_52_O(S)__*_cis_*^-^**

E = -2931.72453530 a.u.

Co -0.13989100 0.13586100 -0.69641800

S -1.60888200 -1.42987200 -0.37671900

S 1.95599000 -1.49820300 -0.77038200

O 1.34054100 1.46459000 -0.34171900

O -1.67078800 1.49321600 -0.53988000

C -2.76473600 0.97827700 -0.16163400

C -2.94805800 -0.38753500 0.05478800

C 2.51062100 1.07200700 -0.05170200

C 2.99370800 -0.22951400 -0.10707400

C -4.12546900 -0.95091800 0.60386900

N -5.08174200 -1.44223300 1.05218700

C -3.88981500 1.88723300 0.06400200

N -4.76213900 2.63215200 0.23058700

C 3.44118300 2.12165300 0.38884900

N 4.14380900 2.97602300 0.73271100

C 4.30985700 -0.57706300 0.28954400

N 5.37784600 -0.90455000 0.61928000

C 1.08157900 -2.12365000 0.71871900

C -0.01909400 -1.17763300 1.14799600

H 1.81853300 -2.28804600 1.51188500

H 0.67280900 -3.09638200 0.42683100

H 0.35429600 -0.21841800 1.52199500

H -0.66990900 -1.60535100 1.90933700

**^3^2y__O(S)_^-^**

E = -2931.76255030 a.u.

Co 0.00023500 -0.96541500 -0.00245700

S -1.26165000 0.71851200 -1.15951500

S 1.26145400 0.71321500 1.16308100

C 2.80792300 -0.83857800 -0.42519400

C 2.81137800 0.25002000 0.44339200

C -2.81132500 0.25137300 -0.44181300

C -2.80739600 -0.84145100 0.42144000

C -0.75265200 2.14972900 -0.11230800

C 0.75220800 2.14932600 0.12273500

H -1.30497100 2.07375700 0.82809000

H -1.04961600 3.07381900 -0.61581100

H 1.04897900 3.07106100 0.63064700

H 1.30454300 2.07796000 -0.81801600

C -3.98008800 0.96629900 -0.79510500

C -4.09585100 -1.27462200 0.98176900

C 4.09662100 -1.26865100 -0.98735100

C 3.97986500 0.96358500 0.80034800

N -4.91458400 1.59322800 -1.09828500

N -5.08805000 -1.64599700 1.45109200

N 4.91432200 1.58912200 1.10650800

N 5.08904400 -1.63743300 -1.45824000

O 1.79694300 -1.51153100 -0.77986000

O -1.79615800 -1.51588500 0.77253600

**^3^TS_2y_O(S)_B_^-^**

E = -2931.71975434 a.u.

Co -0.08261600 -0.68959000 -0.02610100

S 1.36944300 0.85251400 1.14844800

S -1.36361200 0.76638500 -1.19016200

O -1.66660300 -1.26222800 0.94440900

O 1.59703100 -1.52974900 -0.70301700

C -2.78356000 -0.78614800 0.50568800

C -2.86105600 0.15478700 -0.51007100

C 2.83063300 0.17506700 0.40753600

C 2.68914700 -0.94776600 -0.39438300

C 4.08561800 0.76136400 0.70775300

C 3.90545100 -1.55173800 -0.94684600

C -3.99192800 -1.28912100 1.13224500

C -4.09739500 0.68742300 -0.96343900

N 5.09420400 1.28256600 0.96522900

N 4.84442100 -2.05355100 -1.40315500

N -5.09492600 1.14183700 -1.35506500

N -4.94013100 -1.70851200 1.65382400

C 1.01996700 2.34929400 0.11441000

C -0.45165700 2.61957800 0.01258400

H 1.46603000 2.17185200 -0.86914000

H 1.56911000 3.16990100 0.59323600

H -0.70960100 3.45292100 -0.63771300

H -1.02791500 2.58488800 0.93594100

**^3^2y__O(S)_Int_^-^**

E = -2931.73874770 a.u.

Co -0.15425100 -0.35060400 -0.07828700

S 1.56408500 0.92193600 1.08586700

S -1.61132000 0.82582600 -1.36592000

C -2.78260600 -0.74622600 0.52449200

C -2.99788000 0.12073600 -0.51695700

C 2.87996200 -0.02374400 0.37383200

C 2.54162900 -1.07047100 -0.46963100

C 1.58669800 2.49705600 0.03569400

C 0.51104800 3.39661600 0.47264000

H 1.46284800 2.16713700 -0.99770500

H 2.58496500 2.91475100 0.18402600

H -0.49270300 3.25895700 0.08444400

H 0.67357900 4.08075700 1.29855100

C 4.21497600 0.31651800 0.70518400

C 3.62533500 -1.88357400 -1.02575900

C -3.89805700 -1.33428500 1.23933500

C -4.30868700 0.47761600 -0.93738100

N 5.29433600 0.64628300 0.98982400

N 4.46336600 -2.53933400 -1.48277300

N -5.36875000 0.79190700 -1.29991600

N -4.76898900 -1.81850500 1.83413000

O -1.59233000 -1.09058700 0.94364200

O 1.36078600 -1.40968300 -0.80937600

**^3^TS_2y_O(S)_A_^-^**

E = -2931.73296265 a.u.

Co -0.35297400 -0.32558100 -0.04649800

S 1.14886100 0.99629600 1.27568500

S -1.89154200 0.82824800 -1.25016900

C -2.92815500 -1.14042100 0.31504200

C -3.20790500 -0.17471900 -0.61867300

C 2.57223100 0.18170800 0.68840800

C 2.41205800 -0.80269400 -0.28640900

C 1.08999800 2.74745200 -0.08776700

C 2.10449100 3.61373600 0.31470300

H 0.05428800 3.00383400 0.12490800

H 1.22714400 2.18684600 -1.00912100

H 1.93697900 4.33660300 1.10623300

H 3.11870600 3.49635500 -0.05241200

C 3.84583800 0.58242200 1.17003800

C 3.60438000 -1.47487000 -0.80133500

C -3.98472300 -1.96940700 0.86083200

C -4.53024300 0.05553000 -1.08826200

N 4.87216300 0.95498900 1.57232900

N 4.53391800 -2.01687500 -1.23031500

N -5.60192300 0.26846500 -1.48852100

N -4.80839300 -2.64620500 1.31941500

O -1.72605200 -1.37536100 0.77343800

O 1.29889500 -1.17727000 -0.77359400

**^1^1__Cu_OS__*_cis_*^-^**

E = -3110.83845165 a.u.

Cu 0.00045700 0.51082900 0.00089200

S 1.21203900 -1.09339400 -0.83968800

C 2.69708700 -0.37123200 -0.26422900

C 2.66607200 0.96524700 0.11954800

C 3.87877100 -1.15713200 -0.20289100

N 4.83620900 -1.81699900 -0.16747900

C 3.91273800 1.68183600 0.35157800

N 4.89095700 2.27473700 0.53902700

O 1.58207600 1.61959000 0.29742200

C -2.66470000 0.96869000 -0.11513100

C -2.69707000 -0.37024800 0.25982700

C -3.91060200 1.68807800 -0.34244600

C -3.87955400 -1.15453800 0.19354500

N -4.88817600 2.28325500 -0.52600500

N -4.83759200 -1.81330200 0.15405800

O -1.57998800 1.62302000 -0.28872900

S -1.21270900 -1.09773800 0.83045000

**^1^TS_15_Cu_OS__*_cis_*^-^**

E = -3189.39534236 a.u.

Cu 0.16299800 0.08364700 -0.42428500

S -1.61474000 -1.48836500 -0.33638600

C -2.87600500 -0.30079600 -0.25403000

C -2.49250300 1.04150000 -0.30906200

S 1.75910800 -1.42159100 -0.84727200

C 3.06039600 -0.36750400 -0.27456200

C 2.71748100 0.89364000 0.13524100

C -3.53775500 2.06640100 -0.26233000

N -4.34601800 2.89519300 -0.22282500

C -4.22309000 -0.71484100 -0.08925300

N -5.31384200 -1.09154000 0.06153800

C 4.39699100 -0.84595900 -0.26602500

N 5.48161700 -1.26811900 -0.26694800

C 3.73270500 1.83363800 0.57175100

N 4.52408700 2.60219100 0.93135300

O 1.48846900 1.32589300 0.16598900

O -1.30095200 1.45515300 -0.40518600

C -1.21787100 -1.93021800 1.80647100

H -2.22756500 -2.26405800 2.03195900

H -0.50223800 -2.72143300 1.59436500

C -0.74538500 -0.76747700 2.41083200

H 0.31276000 -0.52439300 2.39832900

H -1.43061200 -0.01932300 2.79568900

**^1^5__Cu_OS__*_cis_*^-^**

E = -3189.39487388 a.u.

Cu 0.23125200 -0.56818000 -0.07315300

S -1.71694500 -0.92254500 -1.15408200

C -2.78611800 0.18339400 -0.29972500

C -2.26908300 0.94739000 0.76331700

S 1.32543900 0.85637400 -1.43177700

C 2.82617200 0.65883500 -0.52548800

C 2.83784800 -0.21009200 0.52616100

C -3.21551500 1.88312300 1.40793200

N -3.91934100 2.62156500 1.95645500

C -4.13908600 0.18729200 -0.70273300

N -5.24974700 0.14797900 -1.05513700

C 3.99129600 1.37149600 -0.92581000

N 4.92923200 1.96272900 -1.27689200

C 4.04579000 -0.50262500 1.26281800

N 5.00069400 -0.76320700 1.86917900

O 1.76625600 -0.85851100 0.94103900

O -1.11342800 0.90082700 1.21735100

C -1.73065200 -2.44812900 -0.08370500

H -2.71356400 -2.54042300 0.38125300

H -1.53951300 -3.30128600 -0.73809800

C -0.58411200 -2.11218600 0.82387000

H 0.24529700 -2.81534800 0.86099500

H -0.84216300 -1.67995800 1.78886800

**^1^TS_52_Cu_OS__*_cis_*^-^**

E = -3189.41509237 a.u.

Cu -0.24462300 0.27353000 -0.32152400

S -1.69762200 -1.27873300 -0.44328600

C -3.05687800 -0.26000600 -0.02209100

C -2.87884500 1.12174300 -0.10225400

S 2.11808300 -1.77247700 -0.62155800

C 3.04877300 -0.38083900 -0.05111700

C 2.55966900 0.91299400 -0.17026500

C -4.04236200 1.99125400 0.08181000

N -4.94151300 2.70974600 0.21892300

C -4.26261600 -0.86423800 0.41507200

N -5.24166500 -1.38734200 0.76688500

C 4.35077300 -0.63081900 0.46355300

N 5.40536800 -0.87940800 0.88983500

C 3.47904400 2.01145100 0.16669500

N 4.16740000 2.90863600 0.41857800

O 1.40039300 1.28791200 -0.53215200

O -1.76158300 1.68609800 -0.30371900

C 0.13428800 -1.01012000 1.21264400

C 1.05132600 -2.14082000 0.82077600

H 0.68333000 -0.14167400 1.60390200

H -0.63334700 -1.30069000 1.92559300

H 0.48058500 -3.02933600 0.53432200

H 1.69557300 -2.40723300 1.66692400

**^1^TS_53_Cu_OS__*_cis_*^-^**

E = -3189.35616032 a.u.

Cu -0.21595200 0.22346600 -0.64460600

C 2.71972800 0.74160900 -0.22453000

S 2.03062100 -1.84302100 0.26305300

C 3.17172300 -0.53577200 -0.01313300

C -2.81892000 0.96620700 0.05639200

C -3.02444800 -0.36063400 -0.28142800

C 4.56261600 -0.84421000 -0.01818600

N 5.68880800 -1.12915200 -0.02433700

C 3.66533800 1.82891500 -0.40000100

N 4.40186300 2.71058900 -0.55107000

C -3.94482300 1.74021100 0.57483300

N -4.79708000 2.40402200 1.00028800

C -4.29704800 -0.97220900 -0.12503600

N -5.32603400 -1.50522400 -0.00350200

C 0.97643200 -1.12713800 1.59677500

C 0.65894700 0.32187000 1.47229100

H 1.35048100 1.00826900 1.94706900

H -0.38051100 0.64253200 1.54023700

H 1.50745800 -1.28947200 2.54165700

H 0.05995100 -1.72257600 1.53618000

O 1.46997600 1.10878700 -0.18754000

S -1.69494400 -1.35613300 -0.91110500

O -1.69699000 1.59377600 -0.02380000

**^1^3__Cu_OS__*_broken_*^-^**

E = -3189.43215778 a.u.

Cu -0.45450900 -0.89547600 -0.4904260

S 1.46217100 -0.24917000 -1.39808800

C 2.77220200 -0.30939900 -0.19603200

C 3.12038400 0.74179200 0.59064600

S -2.12563300 -1.95821000 0.42764300

C -3.13694300 -0.49506700 0.41695100

C -2.66812500 0.70430000 -0.07214300

C 4.27603200 0.66815200 1.44722400

N 5.20971900 0.62842800 2.13166400

C 3.50932900 -1.52754700 -0.14332600

N 4.09746000 -2.52762200 -0.13137700

C -4.46053500 -0.59693300 0.93071300

N -5.53863700 -0.70925200 1.35695600

C -3.52882300 1.87728300 -0.06098400

N -4.15196200 2.85789900 -0.08262300

C 1.24600500 2.09814000 0.02064800

C 1.24551400 1.56096700 -1.39616600

H 2.03131900 2.01354100 -2.00552900

H 0.25687100 1.73226700 -1.82759900

H 0.46742900 1.60753300 0.60994700

H 1.07657300 3.17487900 0.02396500

O 2.52635600 1.93660400 0.65957200

O -1.47665000 0.89869200 -0.55942800

**^1^2__Cu_OS__*_cis_*^-^**

E = -3189.45786940 a.u.

Cu -0.02384600 0.52965000 -1.09859500

C -2.43559700 1.16247900 0.31977000

S -1.66235300 -1.07734400 -1.03100800

C -2.78210800 -0.14697800 -0.02154100

C 2.67292600 0.79000300 -0.28567700

S 1.72217800 -1.78440800 -0.25645000

C 2.77346300 -0.47637600 0.28567600

C -4.00022300 -0.76017100 0.35154700

N -4.97716100 -1.31931100 0.65467900

C -3.41956600 1.92570100 1.10902800

N -4.15883700 2.56603400 1.73014600

C 3.72912200 1.75954800 0.05377500

N 4.53922300 2.55628000 0.28030600

C 3.79191600 -0.78955900 1.22448400

N 4.60448600 -1.08622600 2.00470800

C 0.30464100 -1.79274400 0.91525200

C -0.94466900 -2.27004600 0.17899900

H 0.16305800 -0.78911500 1.32583700

H 0.52247500 -2.47625100 1.74134800

H -1.74679600 -2.50293400 0.88446900

H -0.73450200 -3.17931700 -0.39309800

O 1.79314800 1.22796000 -1.08541100

O -1.37606600 1.76544400 0.01332200

**^1^TS_2y_Cu_OS__*_cis_*^-^**

E = -3189.37950316 a.u.

Cu 0.00000100 -0.80619800 0.00000400

S 1.27044700 0.49228500 1.37770900

S -1.27044900 0.49225900 -1.37772200

C -2.71185500 -0.80802900 0.49468900

C -2.74761300 0.07860800 -0.59741100

C 2.74761100 0.07861900 0.59740900

C 2.71185700 -0.80804400 -0.49467000

C 0.48427300 2.82239000 0.48400800

C -0.48427300 2.82238200 -0.48404800

H 1.52780700 2.96188700 0.22248800

H 0.22027200 2.97192200 1.52499500

H -0.22027200 2.97190300 -1.52503700

H -1.52780800 2.96187800 -0.22253100

C 3.97430800 0.64530900 1.04513900

C 3.93384300 -1.03657500 -1.26354200

C -3.93383900 -1.03654400 1.26356800

C -3.97431300 0.64527900 -1.04515800

N 4.96039400 1.13266900 1.42449200

N 4.88306500 -1.23827100 -1.89779300

N -4.96040200 1.13262200 -1.42452500

N -4.88306000 -1.23822700 1.89782600

O -1.68279400 -1.44889300 0.86010400

O 1.68279800 -1.44891800 -0.86007200

**^1^2y__Cu_OS_^-^**

E = -3189.46679469 a.u.

Cu 0.00000000 -0.94892700 -0.00000200

S 1.30420900 0.83129300 1.20714000

S -1.30421100 0.83129300 -1.20714200

C -2.79285900 -0.84924700 0.34202300

C -2.81233500 0.28690500 -0.46736500

C 2.81233500 0.28690500 0.46736600

C 2.79286000 -0.84924600 -0.34202200

C 0.75692600 2.19655600 0.09738400

C -0.75692700 2.19655600 -0.09738700

H 1.28394400 2.07726100 -0.85311800

H 1.06722000 3.14530700 0.54354300

H -1.06722100 3.14530700 -0.54354700

H -1.28394500 2.07726200 0.85311500

C 3.99991400 1.00561500 0.75061800

C 4.07909700 -1.29980800 -0.89880800

C -4.07909500 -1.29980900 0.89881100

C -3.99991400 1.00561400 -0.75061500

N 4.94999000 1.63483300 0.99409600

N 5.06557100 -1.69029400 -1.36438400

N -4.94999100 1.63483300 -0.99409100

N -5.06556800 -1.69029500 1.36438800

O -1.79098800 -1.55196400 0.65283800

O 1.79098900 -1.55196300 -0.65283900

**^1^TS_3_Cu_OS__*_cis_*^-^**

E = -3189.35945787 a.u.

Cu -0.35844200 -0.58700700 -0.43788700

S 1.55373000 -0.26838300 -1.33967700

S -1.97394200 -1.96138200 0.12062900

O 1.63376700 0.71583200 1.39463100

O -1.52583800 1.00635200 -0.34345800

C 2.86569000 -0.38611300 -0.25486600

C 2.68225600 0.13559700 1.04330700

C -3.14002100 -0.62457600 0.23592000

C -2.74288000 0.66643300 -0.01377000

C 3.81281000 0.10563800 1.98146900

N 4.67953900 0.10483700 2.74969600

C 4.15209200 -0.73799300 -0.75064100

N 5.19590300 -1.03306500 -1.16859600

C -4.48488900 -0.92763800 0.58646600

N -5.57855500 -1.20524400 0.87364000

C -3.69264800 1.76026100 0.07880200

N -4.40320100 2.67740300 0.13525000

C 1.16308000 2.36136700 -0.07143100

C 1.39513600 1.95068400 -1.38118900

H 2.33876000 2.19988100 -1.85803600

H 0.51782400 1.89867200 -2.02414600

H 0.14922200 2.32679200 0.31814700

H 1.92940800 2.86717100 0.50381600

**^1^TS_2_Cu_OS__*_cis_*^-^**

E = -3189.43955833 a.u.

Cu 0.34747600 -1.65722300 -0.57296700

S 0.73821300 0.80456100 -0.25867600

S -2.18353600 1.62086300 -1.14261600

O 2.20267500 -1.71879100 -0.17136000

O -1.49770900 -1.50475800 -0.96983900

C 2.90084500 -0.68066500 0.14133700

C 2.44589900 0.62069400 0.18381600

C -2.35341100 -0.99337900 -0.17134900

C -2.78030700 0.32100900 -0.09066900

C 3.24070600 1.73134600 0.53762300

N 3.83979200 2.68977800 0.82102700

C 4.29847400 -0.93660300 0.47223100

N 5.40465700 -1.17414200 0.72610300

C -2.99178000 -1.94999600 0.75382400

N -3.44225000 -2.76215000 1.44759200

C -3.79206400 0.69129900 0.84168500

N -4.59516900 1.06178600 1.59895800

C -1.20150800 2.65632300 0.03296400

C 0.31791500 2.57012400 -0.13559800

H -1.49377200 2.35749300 1.04221500

H -1.52016800 3.69127100 -0.11744600

H 0.81414700 3.01764000 0.72983400

H 0.65059800 3.08684700 -1.03923800
